# Multiple RNA- and DNA-binding proteins exhibit direct transfer of polynucleotides with implications for target-site search

**DOI:** 10.1073/pnas.2220537120

**Published:** 2023-06-20

**Authors:** Wayne O. Hemphill, Calvin K. Voong, Regan Fenske, James A. Goodrich, Thomas R. Cech

**Affiliations:** ^a^Department of Biochemistry, University of Colorado Boulder, Boulder, CO 80309; ^b^BioFrontiers Institute, University of Colorado Boulder, Boulder, CO 80309; ^c^HHMI, University of Colorado Boulder, Boulder, CO 80309

**Keywords:** nucleic acid, chromatin, single-molecule, exchange, displacement

## Abstract

We previously demonstrated that the polycomb repressive complex 2 chromatin–modifying enzyme can directly transfer between RNA and DNA without a free-enzyme intermediate state. Simulations suggested that such a direct transfer mechanism may be generally necessary for RNA to recruit proteins to chromatin, but the prevalence of direct transfer capability is unknown. Herein, we used fluorescence polarization assays and observed direct transfer for several well-characterized nucleic acid–binding proteins: three-prime repair exonuclease 1, heterogeneous nuclear ribonucleoprotein U, Fem-3-binding factor 2, and MS2 bacteriophage coat protein. For TREX1, the direct transfer mechanism was additionally observed in single-molecule assays, and the data suggest that direct transfer occurs through an unstable ternary intermediate with partially associated polynucleotides. Generally, direct transfer could allow many DNA- and RNA-binding proteins to conduct a one-dimensional search for their target sites. Furthermore, proteins that bind both RNA and DNA might be capable of readily translocating between those ligands.

## Significance

Classically, the lifetime of a protein–ligand complex is presumed to be an intrinsic property, unaffected by competitor molecules in free solution. By contrast, a few oligomeric nucleic acid–binding proteins have been observed to exchange competing ligands in their binding sites, and consequently their lifetimes decrease with competitor concentration. Our findings suggest that this “direct transfer” capability may be a more general property of nucleic acid– binding proteins. Thus, many DNA- and RNA-binding proteins could reduce the dimensionality of their search for their target sites by direct transfer to nucleosome DNA, instead of relying entirely on three-dimensional diffusion. Furthermore, direct transfer from nascent RNA to DNA may explain why so many DNA-binding proteins also bind RNA.

A few well-characterized oligomeric proteins have been shown to exchange ligand species through highly unstable ternary complex intermediates (1–5). This mechanism of molecular exchange between protein and polynucleotide molecules has been previously termed “direct transfer,” “facilitated dissociation” (FD), “facilitated exchange,” and “monkey bar mechanism;” herein, we use “direct transfer” since it is the earliest term used to describe direct protein translocation between ligand molecules (1, 6–12). In concurrent studies (13), we demonstrated that direct transfer occurs between RNA and DNA for the chromatin-modifying enzyme polycomb repressive complex 2 (PRC2) (14); this phenomenon could allow mutually antagonistic RNA and DNA binding to both positively and negatively regulate PRC2 activity depending on the transcriptional environment. The prevalence of direct transfer among RNA- and DNA-binding proteins is not known. Thus, a robust study of direct transfer with various nucleic acid–binding proteins (NBPs), including monomeric proteins, was warranted.

NBPs with well-characterized biochemistry span a wide range of biological functions. Heterogeneous nuclear ribonucleoprotein U (hnRNP-U) is an RNA- and DNA-binding protein proposed to regulate chromatin structure and pre-mRNA processing (15). Recent work has also demonstrated that hnRNP-U has specificity for G-quadruplex (G4) RNA (16), much like PRC2 (17). Three-prime repair exonuclease 1 (TREX1) (18, 19) is a 3′-to-5′ exonuclease (20) that degrades DNA to prevent aberrant nucleic acid sensing (21) and the resulting autoimmunity (22). In recent years, interest in TREX1 has risen due to its potential as a cancer immunotherapy target (23), and TREX1 activity on ss- versus dsDNA, the purpose of its homodimer structure, and the source of TREX1’s DNA substrates in vivo remain areas of active interest (24, 25). Fem-3-binding factor 2 (FBF-2) is a Pumilio Factor (PUF) family sequence–specific RNA-binding protein that binds the 3′-untranslated regions of mRNAs to inhibit expression of proteins necessary for meiotic entry during *Caenorhabditis elegans* germline development (26–28). MS2 coat protein (MS2-CP) forms the capsid of this *Escherichia coli* bacteriophage, and it also negatively regulates MS2 replicase expression (29, 30). These functions require MS2-CP binding specifically to a hairpin RNA (31–34).

**Fig. 1.**
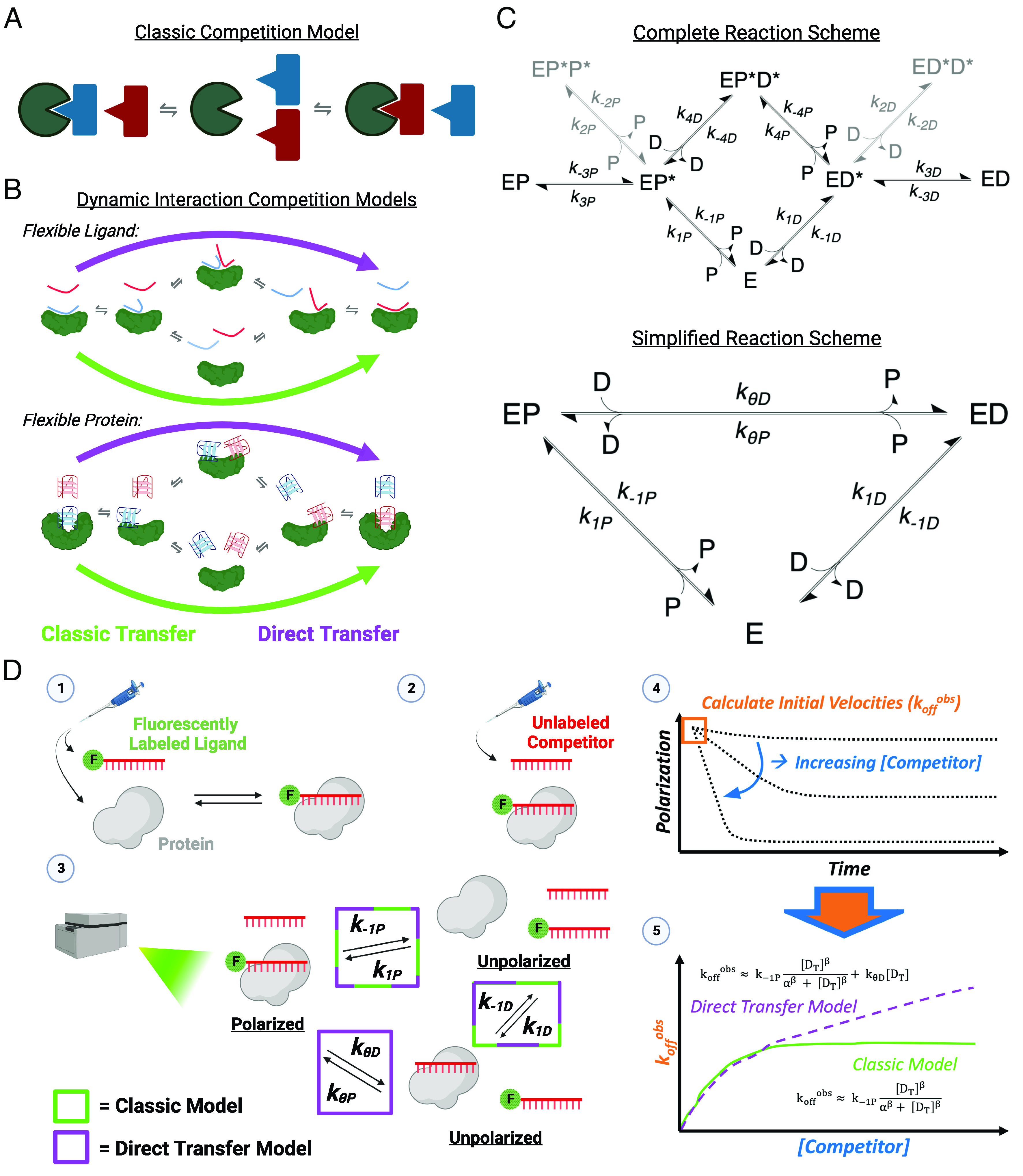
Models and reaction schemes for protein–polynucleotide binding competition. (*A*) Classic model of protein–polynucleotide binding competition. The initial complex fully dissociates at a rate independent of free competitor, then the resulting free protein (green shape) can be bound opportunistically by a competitor polynucleotide (red shape), which prevents rebinding of the original ligand (blue shape). (*B*) Dynamic models of protein–polynucleotide binding competition. Many nucleic acid–binding reactions involve flexible ligands (“Flexible Ligand”) and/or proteins (“Flexible Protein”) and extensive protein–ligand contacts, which might be better described by a dynamic model that allows the formation of short-lived ternary complexes and partial ligand interactions. As a result, a competitor (red shape) can influence how quickly protein (green shape) dissociates from an initially bound ligand (blue shape). We term these “direct transfer” models (purple), in contrast to a classic transfer model (green). (*C*) Reaction schemes for classic and direct transfer. The models in panel *B* can be more agnostically presented as a reaction scheme (Complete). Complete reaction scheme includes protein (E), competitor (D), ligand (P), and numerous rate constants (k), where conjugations of reactants are complexes, and asterisked complex components are partially associated with protein (*SI Appendix*, Eq. **1**). If partially associated complexes are presumed to be highly transient, then the simplified reaction scheme can describe the concentrations of remaining reactants and stable complexes (*SI Appendix*, Eq. **2**). This is the reaction scheme used to experimentally determine the associated rate constant values herein, and its nomenclature corresponds with panel *D* and Table 1; in the cases where competitor and ligand are the same, the P/D nomenclature for rate constants can be omitted. (*D*) Experimental strategy to measure direct transfer kinetics. 1) The minimum amount of protein required for saturated binding is mixed with a trace amount of fluorescently labeled oligonucleotide, then incubated until thermal and reaction equilibrium. 2) Various concentrations of unlabeled competitor oligonucleotide are added to the preformed complex to initiate reactions. 3) The time-course reactions are immediately monitored by fluorescence polarization (FP) in a microplate reader. Potential complexes with their polarization states are shown, and they are labeled with rate constants describing intercomplex transitions. Rate constants associated with a classic competition model are indicated by green boxes, and those additionally necessary for a direct transfer model are indicated by a purple box. 4) Polarization signals are normalized to the range in polarization signal across all competitor concentrations to give proportion of initial complex remaining. Normalized polarization signals are plotted versus time and fit with one-phase exponential decay regression. 5) The initial slopes of the regressions (k_off_^obs^) are plotted versus competitor concentration and regressed with custom equations describing the classic competition and direct transfer models to determine rate constant values. Models are compared with the Bayesian Information Criterion (BIC).

Here, we use biophysical assays to interrogate the prevalence, mechanism, and biophysical requisites of direct transfer among these NBPs. Our findings indicate that direct transfer occurs when polynucleotides compete for shared protein contacts and partially associate to form an unstable ternary complex intermediate (Fig. 1 *A*–*C*). This supports direct transfer being a feature of many RNA-binding chromatin-associated proteins that may be generally required for their tunable regulation (13). Notably, prior work has suggested that direct transfer could allow for protein movement along DNA (11, 12, 35–37), which would allow many NBPs to more efficiently search for their gene targets after initial chromatin association (38, 39). Similarly, RNA-binding proteins could be transferred intramolecularly and intermolecularly in search for optimal binding sites. In addition, NBP complexes in vivo can be prematurely displaced by other binders in the reaction environment, instead of being rate limited by intrinsic dissociation of the complex, as previously discussed for FD (6, 40). These implications substantially expand the possibilities for how NBPs find their respective binding partners and regulate their biological activity.

**Table 1.** Rate constants for protein–ligand interactions

Protein	Ligand	Competitor	T (°C)	K_dP_^app^ (nM)	k_−1P_ (s^−1^)	k_θD_ (M^−1^s^−1^)	HOP
hnRNP-U	r(G_3_A_2_)_4_[F]	r(G_3_A_2_)_4_	25	15 ± 4.8	^*^n.d. (≥3.3×10^−2^)	^*^n.d.	n.d.
			4	n.d.	6.4 ± 0.42 (×10^−4^)	39 ± 9.2	−0.98
		^||^r(G_3_A_2_)_4_ | C_1_			^‡^9.7 (×10^−4^)	^‡^35	−1.7
Streptavidin	FAM-Biotin	Biotin	25	^†^2.5 ± 0.16	^¶^1.3 ± 0.68 (×10^−5^)	^§^0	n/a
			37	n.d.	^‡^^,^^¶^1.7 (×10^−5^)	^§^0	n/a
TREX1	[F]d(N)_5_	d(N)_5_	25	8.9 ± 3.8	^‡^^,^^#^7.0 (×10^−3^)	^‡^^,^^#^1,500	0.84
			4	n.d.	6.8 ± 0.93 (×10^−3^)	700 ± 84	−0.22
		^||^d(N)_5_ | C_2_			^‡^6.6 (×10^−3^)	^‡^610	−0.38
		ds-d(N)_60_		n.d.	^‡^6.7 (×10^−3^)	^‡^1,200	−0.58
	ds-[F]d(N)_60_	d(N)_5_	25	30 ± 3.9	^‡^^,^^#^1.9 (×10^−2^)	^‡^^,^^§^0	n/a
			4	n.d.	1.3 ± 0.34 (×10^−2^)	^§^0	n/a
TREX1^R174A,K175A^	[F]d(N)_5_	ds-d(N)_60_	25	46 ± 16	n.d.	n.d.	n.d.
			4	n.d.	^‡^^,^^#^7.1 (×10^−3^)	^‡^^,^^#^330	−1.4
	ds-[F]d(N)_60_	d(N)_5_	25	130 ± 28	n.d.	n.d.	n.d.
			4	n.d.	6.7 ± 2.6 (×10^−3^)	280 ± 65	−1.5
FBF-2	[F]RNA^PUF^	RNA^PUF^	25	50 ± 9.2	4.4 ± 0.42 (×10^−3^)	140 ± 55	1.4
		^||^RNA^PUF^ | C_3_	4→25	n.d.	1.1 ± 0.10 (×10^−3^)	42 ± 13	−1.7
MS2-CP	[F]RNA2^MS2^	RNA1^MS2^	25	^†^2.9 ± 0.62	1.4 ± 2.0 (×10^−3^)	89 ± 51	−0.92
		^||^RNA1^MS2^ | C_3_			^‡^4.6 (×10^−3^)	^‡^57	−3.3
			4	n.d.	^‡^2.1 (×10^−3^)	^‡^46	−2.5

FP-based methodology (Fig. 1*D*) was used to determine the apparent equilibrium dissociation constants (K_dP_^app^), intrinsic dissociation rate constants (k_−1P_), direct transfer rate constants (k_θD_), and “hand-off” proficiency (HOP) scores for several protein–ligand interactions. HOP scores are defined in Theoretical Background (*SI Appendix*) and are a metric of the ratio k_θD_/k_−1P_, where positive or negative values indicate above- or below-average propensity for direct transfer, respectively. Values indicate mean ± SD for at least three independent experiments. K_d_ experiments were carried out in the absence of competitor and were fit with standard (non-Hill, nonquadratic) regression. Rate constant definitions are in Fig. 1*D*, ligand/competitor definitions are in *SI Appendix*, Table S2, and binding curves with standard versus Hill regression are in *SI Appendix*, Fig. S1*A*. n.d. = not determined; n/a = not applicable.

^*^Dissociation completed during initiation-measurement delay (~90 s).

^†^Experiment used [Prey] ≥ K_dP_^app^; it is possible that K_d_ < K_d_^app^.

^‡^Value from single experiment.

^§^Bayesian Information Criterion (BIC) favored classic competition; rate constant not applicable.

^¶^Early partial dissociation curves and manual baseline used for regression; it is possible that k_-1_ < k_–1_^app^ or k_θ_ < k_θ_^app^.

^#^Late partial dissociation curves used for regression; it is possible that k_–1_ > k_-1_^app^ or k_θ_ > k_θ_^app^.

^||^Total polynucleotide concentration was kept constant by serially diluting competitor in a carrier nucleic acid; C_1_ = r(A)_20_, C_2_ = r(N)_5_, and C_3_ = r(A)_10_.

## Results

### Diverse Protein–Polynucleotide Interactions Exhibit Direct Transfer Kinetics.

Based on prior mechanistic proposals for direct transfer (1, 6, 11) and our concurrent PRC2 direct transfer studies (13), we suspected that many NBPs may have direct transfer capability. This could derive from competition on many NBP surfaces being less rigid than posited by a classic model (Fig. 1*A*), and instead being dynamic enough to facilitate direct transfer reactions within a single binding site (Fig. 1*B*). In *SI Appendix*, *Theoretical Background*, we outline how the complete reaction scheme for such a direct transfer model can be well approximated by a simplified reaction scheme (Fig. 1*C*), which others have previously discussed (1). Furthermore, we demonstrate that the rate constants for this simplified reaction scheme can be accurately determined experimentally with FP–based competitive dissociation (FPCD) experiments and regression models (Fig. 1*D* and *SI Appendix*, Table S1).

**Fig. 2.**
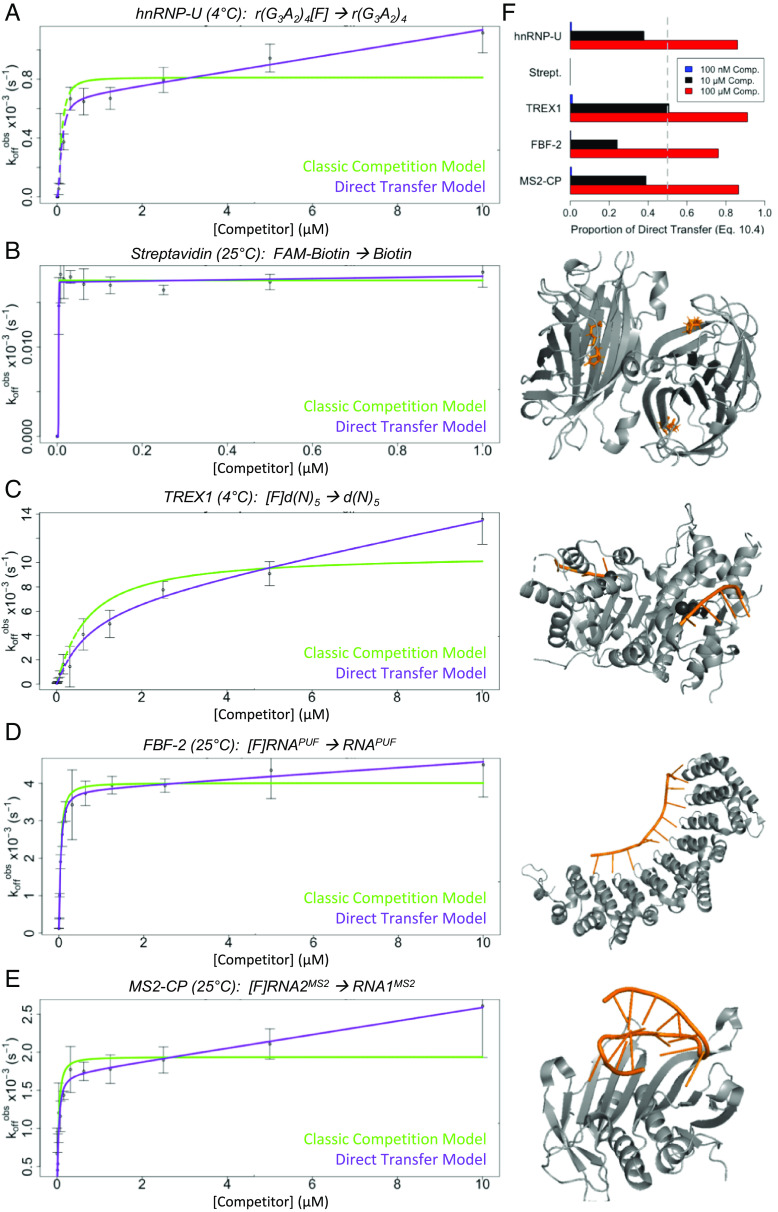
All tested nucleic acid–binding proteins exhibit kinetics consistent with direct transfer. (*A*–*E*) For several protein–ligand interactions, FPCD experiments were performed and analyzed as described (Fig. 1*D*), and the final plots of apparent ligand dissociation rate (k_off_^obs^) versus competitor concentration are shown alongside the crystal structures of their interactions. Plots show best-fit regression of the data, with equations describing classic competition (green lines) versus direct transfer (purple lines). Graphs are from representative experiments of at least 3, and error bars are mean ± SD across four reaction replicates. Crystal structures show proteins as gray cartoons and nucleic acid ligands as orange cartoons/sticks. Structures in panels *B*–*E* are PDB 2IZJ, 2OA8, 3V74, and 2C51, respectively; no crystal structure exists for hnRNP-U. The corresponding regression values are in Table 1, ligand K_d_^app^ and equilibrium competition data are in *SI Appendix*, Fig. S1, raw data examples are in *SI Appendix*, Fig. S2, carrier polynucleotide control experiments are in *SI Appendix*, Fig. S3, helpful nomenclature definitions are in Fig. 1*D*, and ligand/competitor definitions are in *SI Appendix*, Table S2. (*F*) The predicted proportion of protein translocations between polynucleotides proceeding through a direct transfer versus classic pathway (Fig. 1*B*) at various competitor effective molarities (“Comp.”). Data are for the interactions in panels *A*–*E*. Dashed gray line indicates equivalent flux through classic and direct transfer pathways.

To assess the generality of direct transfer, we selected four well-characterized protein–nucleic acid interactions, including hnRNP-U binding to G4 RNA (16), TREX1 (sequence independent) binding to short ssDNA (18, 19), FBF-2 (sequence specific) binding to a short ssRNA sequence (26, 27), and MS2-CP (sequence specific) binding to its RNA hairpin motif (31, 32). We also tested streptavidin binding to biotin as a non-NBP control (41). These five protein–ligand systems were chosen for their collective diversity in structure, type of bonding between protein and ligand, ligand specificity, binding surface area, affinity, and stoichiometry. We used purified recombinant protein and FP experiments to determine K_d_^app^ for these interactions (Table 1 and *SI Appendix*, Fig. S1). Our binding affinity results were generally consistent with prior reports for TREX1 (42), hnRNP-U (16), MS2-CP (33), FBF-2 (27), and streptavidin (41), including the observation that streptavidin and hnRNP-U were more appropriately described by a Hill regression model (*SI Appendix*, Fig. S1*A*). Streptavidin and MS2-CP have significantly lower K_d_ than can be accurately determined by our methodology, so their binding curves are likely limited by ligand concentration (Table 1). Then, we performed FPCD experiments (Fig. 1*D*) with ligand (fluorophore labeled) and competitor (unlabeled) polynucleotides of the same identity to determine which protein–ligand interactions, if any, had an apparent dissociation rate (k_off_^obs^) that was dependent on the competitor concentration (i.e., direct transfer), versus plateauing at high competitor concentrations (i.e., classic competition). In other words, was the dissociation rate sufficiently described by one rate constant for classic dissociation (k_−1_), or did it require an additional rate constant for direct transfer (k_θ_)? Unexpectedly, our results (Fig. 2) revealed direct transfer-like dissociation kinetics for every NBP, with their second-order rate constants for direct transfer spanning a 40-fold range. We note that the direct transfer rate constants are all several orders of magnitude lower than typical association rate constants, which we attribute to partial association of the competitor (to initiate direct transfer) being rate limited by infrequent fluctuation of the bound ligand to a partial association state. Only the streptavidin–biotin interaction had an apparent dissociation rate that plateaued at high competitor concentrations, consistent with classic competition. The equilibrium data from these competition reactions are provided in *SI Appendix*, Fig. S1*B*.

Since it was unexpected that these diverse protein–nucleic acid interactions would all exhibit direct transfer, we considered possible artifactual explanations for the data. First, the assumptions of our regression and analysis approach (*SI Appendix*, *Theoretical Background* and *Methods*) include exponential dissociation for reactions, so nonexponential dissociation of our complexes might produce k_off_^obs^ calculation errors that mimic direct transfer kinetics. However, our raw data clearly exhibited exponential dissociation (*SI Appendix*, Fig. S2). Next, it seemed possible that variations in k_off_^obs^ across competitor concentrations could result from nonspecific polynucleotide concentration–dependent factors, such as polynucleotide aggregation or perturbation of the ionic environment of the reactions. To test this, we repeated the experiments of Fig. 2 using a nonbinding carrier polynucleotide to keep total competitor polynucleotide concentration constant while varying the ratio of binding to nonbinding competitor. We found that all protein–polynucleotide interactions still exhibited direct transfer kinetics (*SI Appendix*, Fig. S3). Collectively, our findings suggest that direct transfer might be widespread among NBPs.

Relative flux through classic versus direct transfer pathways (Fig. 1*B*), as a function of competitor concentration, is described by *SI Appendix*, Eq. **10.4**; this suggests that direct transfer significantly affects our NBPs only at low-micromolar or higher effective concentrations of competitor (Fig. 2*F*). Relative pathway flux is directly related to the ratio k_θ_/k_−1_ for a protein–ligand interaction, and these ratios for our NBPs fall within the range of those for previously interrogated direct transfer proteins such as SSB, CAP, and recA (1–3).

### Single-Molecule (SM) Experiments Support Direct Transfer for TREX1.

As an orthogonal test for direct transfer (Fig. 1*B*), we employed total internal reflection fluorescence (TIRF) microscopy-based SM experiments (43). We chose the TREX1 + 5-mer ssDNA interaction for these experiments because it was well behaved. Although TREX1 is a dimer, the two DNA-binding sites cannot be spanned by DNA molecules of the length tested, and the protein does not exhibit cooperative binding for short ssDNA ligands (24, 42) (*SI Appendix*, Fig. S1*A*). Prior crystal structures indicate that ligands bound to separate protomers should have their 5′-fluorophores separated by ~8 nm with opposing orientations, suggesting that Förster/fluorescence resonance energy transfer (FRET) efficiency between these ligands may be limited (44–46).

**Fig. 3.**
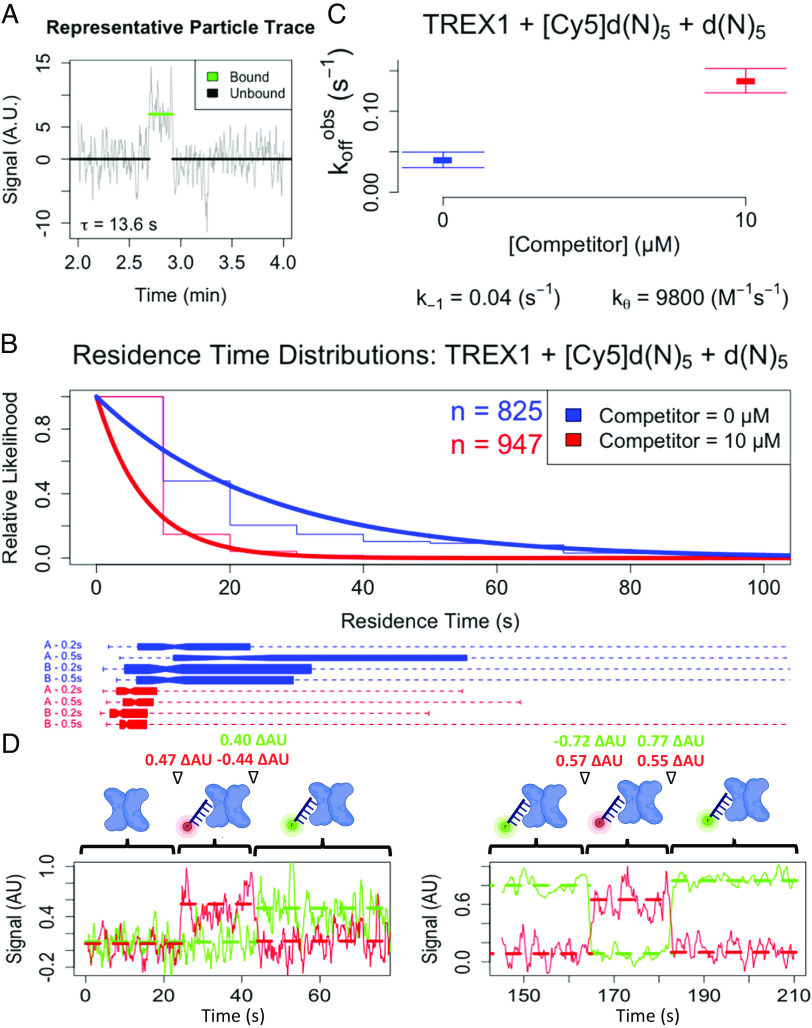
Single-molecule experiments support direct transfer of two DNA molecules on TREX1. (*A*–*C*) Single-molecule TIRF-microscopy experiments used FLAG-tagged TREX1 conjugated to the microscope slide, Cy5-labeled ligand DNA, and unlabeled competitor DNA. (*A*) Representative particle trace of TREX1-binding event. (*B*) Distributions of single-molecule residence times. Slides were prepared with labeled ligand only (blue), or labeled and unlabeled ligands (red), in two independent days of experiments (A and B). On each day and for each condition, at least four replicate movies each were collected with two different power/exposure settings (0.2 s and 0.5 s). All data for each condition were regressed with an exponential probability density function, which is shown normalized alongside a normalized histogram (top graph). Total event numbers recorded for each condition are also shown (n). Box-and-whisker plots (bottom graph) of the distribution of single-particle residence times are shown for each contributing dataset for each condition, which are comprised of at least four replicate movies. On box-and-whisker plots, narrowed box centers indicate median, boxes define innerquartile range, and line segments define total range; top-to-bottom, the plots represent 157, 114, 320, 234, 185, 198, 274, and 290 data points. (*C*) Effect of competitor on apparent TREX1–ssDNA dissociation rate. Apparent dissociation rates (k_off_^obs^) were calculated for each contributing data set in panel *B* via regression with an exponential probability density function and are plotted as mean ± SD. The dissociation (k_−1_) and direct transfer (k_θ_) rate constants were calculated from apparent dissociation rates (k_off_^obs^) as described in *Materials and Methods*. (*D*) Observing direct transfer of ligands on TREX1. SM-TIRF colocalization experiments were carried out using FLAG-tagged TREX1 conjugated to the microscope slide and a mixture of Cy5- (red) and Cy3-labeled (green) ligands. Slides were prepared with 5 to 20 pM of conjugated protein and 1 to 10 nM of each ligand, then respective binding states were simultaneously monitored via dual red and green channel excitation and imaging. Example traces of observed ligand transfer events are shown, taken from two different days of experiments. Cartoons illustrate respective binding states for TREX1 homodimer (blue), Cy3-labeled ligand (green), and Cy5-labeled ligand (red). Solid lines indicate signal, dashed horizontal lines indicate average signal per state, and corresponding changes in average signal between states (ΔAU) are shown at top between corresponding cartoons.

Unlabeled TREX1 was conjugated to a microscope slide, fluorophore-labeled oligonucleotide was flowed onto the slide with or without an excess of unlabeled oligonucleotide, and millisecond-interval movies were recorded to track individual particle-binding events (e.g., Fig. 3*A*). Distributions of residence times across all individual binding events (Fig. 3*B*) were used to calculate k_off_^obs^ for each movie (Fig. 3*C*), and then the average k_off_^obs^ values under each reaction condition were used to approximate k_−1P_ and k_θD_ (Fig. 3*C*). The SM results (k_−1_ ≈ 4.0 × 10^−2^ s^−1^, k_θ_ ≈ 9,800 M^−1^s^−1^) had rate constant values about five-fold higher than those of our corresponding FP data (k_−1_ ≈ 0.7 × 10^−2^ s^−1^, k_θ_ ≈ 1,500 M^−1^s^−1^), but they were performed under different buffer conditions. Importantly, the k_θ_/k_−1_ ratios between SM and FP results were similar (2.4 × 10^5^ M^−1^ versus 2.1 × 10^5^ M^−1^), and the competitor dependence of residence times was quite evident (Figs. 2 and 3*B*). Photobleaching control experiments indicated that only 5-20% of these apparent dissociation events were attributable to photobleaching (*Materials and Methods*). These findings demonstrate that the observation of direct transfer kinetics for TREX1 is not restricted to FP-based methodology.

To directly observe transfer events, we modified the initial SM experiments to simultaneously track the TREX1-binding states of 5-mer ssDNA labeled with two different fluorophores. Across two such independent experiments, we identified n = 36 (10 nM of each fluorescently labeled ligand) or n = 34 (1 nM each ligand) apparent direct transfer events among 453 (10 nM each ligand) or 781 (1 nM each ligand) binding events (Fig. 3*D*); such modest proportions (7.9% or 4.4%, respectively) of direct transfer versus classic binding events were expected under the low ligand concentrations necessary for these experiments. We declare the limitation that we cannot statistically associate the relative frequency of these observed ligand exchange events with increasing free ligand concentration, since higher fluorophore concentrations cannot be used in these SM experiments. We observed no instances in these experiments of more than two ligand molecules stably binding a TREX1 homodimer, suggesting that two ligands do not stably bind a single TREX1 protomer. To further interrogate this, we also recorded movies under conditions to detect FRET, and we were unable to detect any stable FRET signals (red/acceptor channel) on the same slides and fields of view as the colocalization experiments despite comparable particle numbers in the green/donor channel (raw data available; see Data, Materials, and Software Availability). Collectively, these findings suggest that TREX1 protomers can be directly transferred between ligands, likely via a short-lived (i.e., <150 ms) ternary intermediate.

### Nonmultimeric Interactions Can Exhibit Direct Transfer Kinetics.

Previous reports of direct transfer have concerned multimeric complexes with higher protomer–polynucleotide binding ratios, where ternary intermediates can be achieved by separate protomers binding separate ligands (1–3, 7). However, existing crystal structures of TREX1 (44), FBF-2 (27), and MS2-CP (47) bound to ligand reveal that only one active site contributes to binding of each ligand, suggesting that their direct transfer must be occurring within a single protomer binding site. To confirm this implication, we performed FP-based stoichiometry experiments (*SI Appendix*, *Supplemental Methods*) to determine the number of ligand molecules bound to each functional unit of our proteins. Our results for all protein–polynucleotide interactions (*SI Appendix*, Fig. S4) were consistent with the stoichiometry expected from their crystal structures. These findings indicate that direct transfer can occur for protein–polynucleotide interactions within a single protomer binding site.

### The Direct Transfer Mechanism Is Revealed in TREX1 Ligand Competition.

**Fig. 4.**
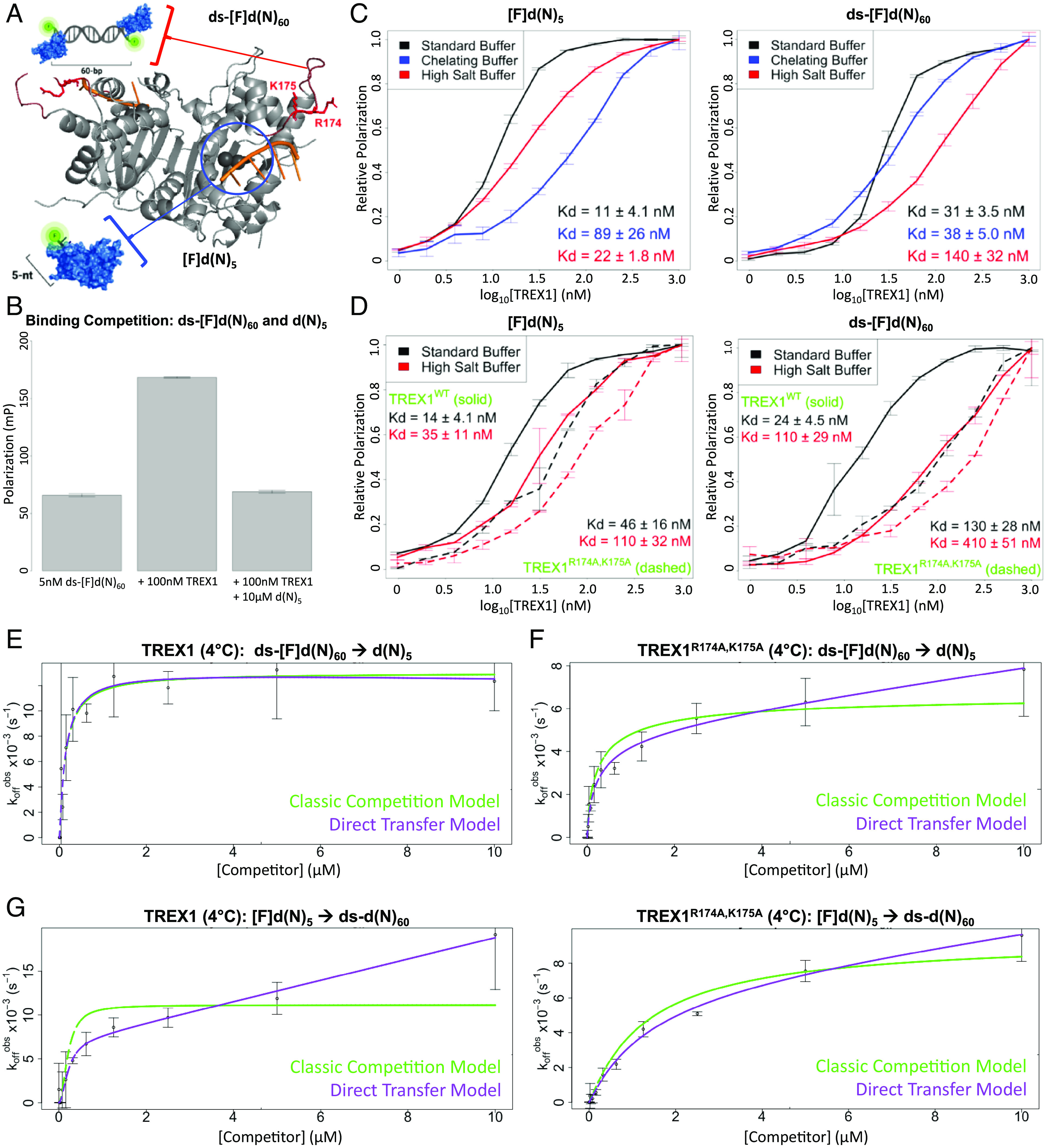
Two competing TREX1 ligands exhibit different magnitudes of direct transfer. (*A*) TREX1 binds long dsDNA with extra protein–polynucleotide contacts. TREX1 (gray cartoon; ref. 24) binds exposed 3′-hydroxyls of DNA (orange cartoon) in its nucleotide-binding pocket (blue circle) with the help of divalent metal ions (black spheres). TREX1 makes additional contacts with long dsDNA through its flexible binding loop (dark red cartoon), particularly residues R174 and K175 (red sticks, labeled). We designed a 5-nt ssDNA that exclusively binds TREX1’s nucleotide-binding pocket ([F]d(N)_5_), and a 60-bp dsDNA having significant additional interactions with TREX1’s flexible binding loop (ds-[F]d(N)_60_). (24). (*B*) Two TREX1 ligands exhibit competitive binding. Fluorescence polarization was measured for labeled 60-bp dsDNA alone (5 nM ds-[F]d(N)_60_), TREX1 with labeled 60-bp dsDNA (+100 nM TREX1), and TREX1 with labeled 60-bp dsDNA and unlabeled 5-nt ssDNA (+100 nM TREX1 +10 µM d(N)_5_). Plot bars indicate mean ± SD from 8 reaction replicates. (*C*) Effects of buffer conditions on K_d_^app^ for two ligands binding to TREX1. Graphs are composites of three experiments with at least two reaction replicates each, where error bars indicate mean ± SD, and K_d_ values are mean ± SD across the replicate experiments. (*D*) Effects of flexible binding loop mutations on K_d_^app^ for each ligand. Standard binding experiments with TREX1 (solid lines) and TREX1^R174A,K17A^ (dashed lines) were performed for the indicated ligands and binding buffers. Number of replicates and error bars as in (*C*). (*E*–*G*) Direct transfer for the TREX1 ds[F]d(N)_60_ and d(N)_5_ ligands is tunable. FPCD experiments (Fig. 1*D*) were performed for the indicated substrates, and the data fit with equations for classic competition (green line) and direct transfer (purple line). Graphs are from representative experiments (n ≥ 3 for panels *E* and *F*, n = 1 for panel *G*), where error bars are mean ± SD across four technical replicates.

Direct transfer was ubiquitous among our tested NBPs (Fig. 2) with virtually identical ligand and competitor molecules, implicating shared protein contacts in the direct transfer mechanism. Consequently, we suspected that NBPs might accommodate direct transfer due to intrinsically dynamic protein–polynucleotide binding interfaces (Fig. 1*B*). This model for the direct transfer mechanism suggests that its kinetics are likely influenced by the proportion of shared versus unshared protein contacts for competing ligands. TREX1 has well-characterized properties that make it suitable for interrogating this hypothesis. Existing crystal structures demonstrate that the 3′-termini of short ssDNA (44) and lengthier dsDNA (48) are positioned identically in TREX1’s nucleotide-binding pocket (Fig. 4*A*), and biochemical studies indicate that lengthy dsDNA (>10 bp) has significant additional interactions with TREX1’s flexible binding loop (Fig. 4*A*) that do not occur for short ssDNA (24, 49). Thus, we designed two different ligands for TREX1 (Fig. 4*A*): a ssDNA 5-mer, [F]d(N)_5_, which should only bind in TREX1’s nucleotide-binding pocket, and a 60-bp dsDNA, ds-[F]d(N)_60_, which should bind in TREX1’s nucleotide-binding pocket with an additional “foothold” on its flexible binding loop. We anticipated that the dsDNA ligand’s foothold on TREX1’s flexible binding loop would prevent efficient direct transfer of TREX1 from the dsDNA ligand to the ssDNA ligand, while direct transfer of ssDNA to dsDNA should occur.

We confirmed competitive binding of the two DNAs to TREX1 via FP (Fig. 4*B*). Next, we tested the proposed binding interactions for the two ligands. Protein–ligand interactions in the TREX1’s nucleotide-binding pocket occur primarily through its two divalent metal ions and hydrogen bonding with 3′-terminal nucleotides (44), while interactions with its flexible binding loop are reportedly via ionic bonding with the DNA backbone (24). We measured TREX1 binding to both ligands by FP. TREX1’s binding affinity for the ssDNA ligand was significantly reduced by divalent metal ion chelation and minimally reduced by higher salt concentrations, while its affinity for the dsDNA ligand exhibited the opposite trend (Fig. 4*C*), supporting the binding interactions proposed for these two ligands. To further validate the binding interactions, we introduced the R174A/K175A suppression-of-function mutations (49) (Fig. 4*A*) to TREX1’s flexible binding loop and repeated these binding experiments. The mutant had affinity for the ssDNA ligand comparable to that of the WT protein with no change in salt sensitivity, but it had significantly reduced affinity for the dsDNA ligand with ablated salt sensitivity (Fig. 4*D*). Collectively, these findings confirm that our two TREX1 ligands are competitive binders, that the ssDNA ligand primarily interacts with TREX1’s nucleotide-binding pocket, and that the dsDNA ligand has a substantial additional foothold on TREX1’s flexible binding loop.

To test whether the dsDNA ligand was resistant to displacement by ssDNA, we performed FPCD experiments (TREX1 dsDNAssDNA transfer, Fig. 1*D*). Our results (Fig. 4*E* and Table 1) demonstrated a competitor concentration-independent dissociation rate at high competitor concentrations. This was consistent with classic competition, indicating that direct transfer was ablated (Fig. 2*C*). This is our only example (within this particular study) of a protein–polynucleotide interaction without detectable direct transfer under our experimental conditions. Repeating these experiments with the TREX1 flexible binding loop mutant, which should perturb the dsDNA ligand’s foothold, restored direct transfer kinetics (Fig. 4*F* and Table 1), consistent with our proposed mechanism that the flexible loop acts as an additional foothold for dsDNA to prevent its displacement. Finally, we expected that testing both proteins with ssDNA as a ligand and dsDNA as a competitor would exhibit direct transfer, since the ssDNA has no unique foothold to mitigate direct transfer by the dsDNA. Indeed, our results (Fig. 4*G*) were consistent with direct transfer kinetics for both the wild-type and mutant TREX1 enzymes. These findings support our proposed direct transfer mechanism (Fig. 1*B*).

### Fast Intrinsic Dissociation Is Correlated with Rapid Direct Transfer.

**Fig. 5.**
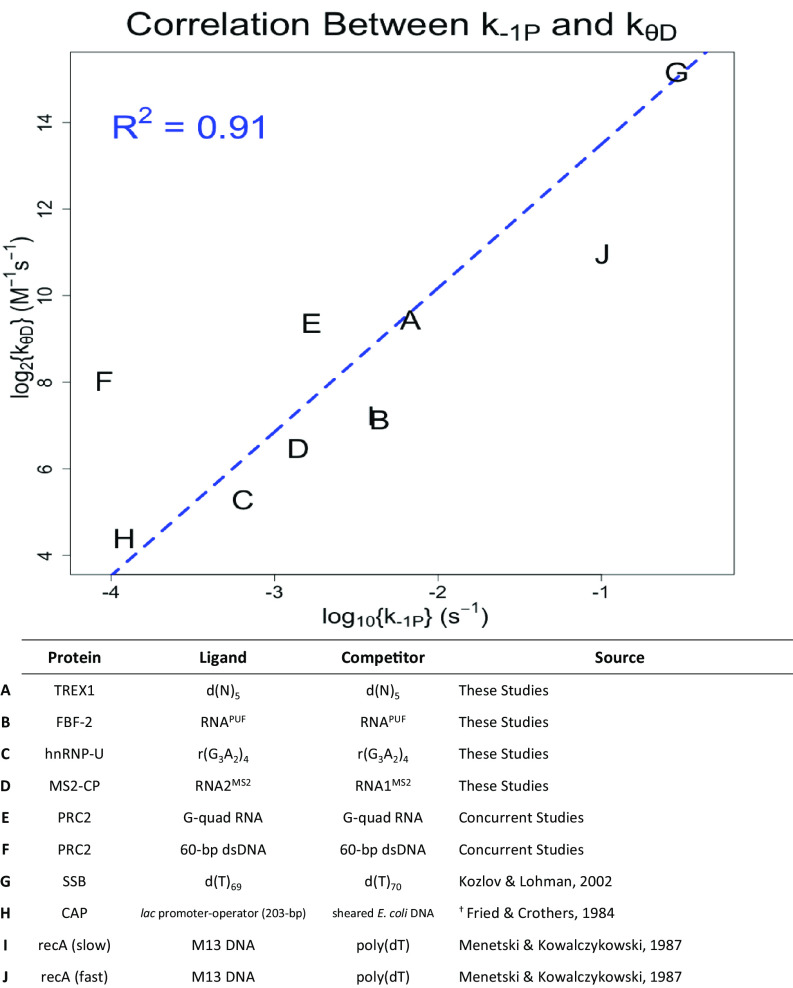
Direct transfer kinetics correlate with intrinsic dissociation kinetics. Direct transfer rate constants (k_θ_) are plotted as a function of intrinsic dissociation rate constants (k_−1_), where rate constant nomenclature follows Fig. 1*D*. Each letter in the graph corresponds to a NBP direct transfer reaction, whose identities are listed in the table below. Regression of the data (blue dashed line) was performed on linear axes with a linear 0-intercept model. Data sourced from these studies refer to the lowest temperature isothermal competition experiments with no carrier polynucleotide, found in Table 1 and Fig. 2, and other data are sourced as described in *SI Appendix*, *Supplemental Methods*. Ligand/competitor definitions for these studies are in *SI Appendix*, Table S2.^†^ Rate constant values were not explicitly stated in source publication; values were calculated from other source data as described in *Materials and Methods*.

Our model for the direct transfer mechanism (Fig. 1*B*) suggests that direct transfer (k_θ_) and dissociation (k_-1_) rate constants should be correlated (*SI Appendix*, Eq. **6.3** and  *Theoretical Background*). To test this implication, we compiled rate constant data from these studies (Table 1), concurrent studies (13), and applicable published studies (1–3) and plotted k_θ_ as a function of k_−1_ (Fig. 5). The results reveal a striking correlation between these rate constants (R^2^ = 0.91), and this correlation was maintained within only this study (R^2^ = 0.75) and only published studies (R^2^ = 0.89). We emphasize that, as noted above (*SI Appendix*, Eq. **10.4** and Fig. 2*F*), an NBP’s location relative to the trendline in a graph shown in Fig. 5 is related to how quickly increasing ligand concentrations lead to increasing flux through a direct transfer versus classic pathway (Fig. 1*B*). Consequently, k_θ_/k_−1_ may be informative of relative direct transfer efficiency, and we propose a hand-off proficiency (HOP) score as a metric (*SI Appendix*, *Theoretical Background*); an HOP score of zero indicates a HOP that is average for the tested proteins. Taken together, these findings support the direct transfer mechanism (Fig. 1*B*) and its consistency among numerous independent studies.

## Discussion

Our surveyed NBPs included RNA- and DNA-binders, single- and double-stranded polynucleotides, disordered and folded polynucleotide structures, variable protein–polynucleotide contact surface areas, and sequence-independent versus sequence-specific binders. Notably, direct transfer occurred for virtually every NBP we studied, although the direct transfer rate constants varied among these interactions (Fig. 5 and Table 1). This implies that direct transfer could be a common capability among NBPs, though definitive conclusions will require further experimental testing. Importantly, we note that direct transfer can occur between identical polynucleotides (i.e., self-competition), so even NBPs with a single preferred ligand can be affected by the consequences of direct transfer.

### Requirements for Direct Transfer.

Direct transfer has been extensively studied for oligomeric NBPs (1–5, 50, 51) and one with separate flexible DNA-binding domains (11). For such proteins, it is intuitive how an incoming polynucleotide could gain a “foothold” on one of several unbound protomers to facilitate its displacement of a second polynucleotide prebound to a different protomer. Our findings (Fig. 2 and *SI Appendix*, Fig. S4) expand the relevance of direct transfer, suggesting that it may be common among RNA- and DNA-binding proteins that interact with a polynucleotide through only one binding site. Our working hypothesis is that direct transfer stems from dynamic protein–ligand contacts in a protein-binding site, as shown in Fig. 1*B*. This implies that direct transfer fundamentally relies on 1) shared protein contacts between competing polynucleotides, 2) the capacity for partial protein–polynucleotide interactions, and 3) the capacity to form transient ternary complexes. In the case of TREX1, our SM experiments showed multiple examples of directly correlated binding of one ssDNA and release of another (Fig. 3*D*) without stable FRET signaling, consistent with a short-lived (e.g., <150 ms) ternary complex. Furthermore, the magnitude of direct transfer (as reflected in k_θ_) should be directly related to the extent of protein–polynucleotide contacts, the proportion of protein contacts shared by two polynucleotides, and the flexibility of the protein–polynucleotide interface(s), and it should be inversely related to the strength of the protein–ligand interaction. The last of these proposals is supported by the correlation between the k_−1_ and k_θ_ rate constants in Fig. 5.

The degree to which each of these properties contributes to direct transfer is an open question. Since streptavidin–biotin epitomizes extremes of these properties and does not exhibit direct transfer (Fig. 2*B*), we expect that there are thresholds for each of these properties, past which direct transfer cannot meaningfully occur. However, we tested high-affinity interactions with RNA hairpins (Fig. 2*E*) and 3 to 4 nt of DNA (Fig. 2*C*), which have very limited protein-binding interfaces, and these still exhibited direct transfer to some degree. Finally, while direct transfer was absent for streptavidin–biotin, we note that it may still occur for other small-molecule binders (12). Our findings suggest requisite properties for direct transfer and provide an initial framework for their quantitative boundaries, but they compel further studies with other protein–polynucleotide interactions to better define the biophysical requirements of direct transfer.

### Considerations for Methodologies to Assess Dissociation Rate Constants and/or Mean Lifetimes.

Recent studies (6–9) of FD, where proteins compete one-another off gene targets through the same mechanism that polynucleotides compete one-another off proteins in direct transfer, have implications that are echoed for direct transfer due to their shared kinetic mechanism. Notably, methodologies that infer dissociation rates through “chase” of preformed complexes with an excess of competitor, such as the FP-based experiments used here (52, 53), should consider that the competitor may not be a neutral participant in ligand dissociation. Making measurements across competitor concentrations may confirm classic competition, but if direct transfer is instead revealed, then extrapolation is required to derive the intrinsic dissociation rate (Fig. 1*D*). Alternatively, methods that do not rely on competitors to prevent reassociation could be considered, such as surface plasmon resonance (SPR) (54). SPR removes dissociated ligand from the reaction via constant buffer flow across a binding surface, which should limit direct transfer. Like “chase” experiments, SM approaches that leave dissociated ligand in the reaction can have their measurements of mean complex lifetime skewed by direct transfer events causing premature complex dissociation, though the effects should be negligible under typical ligand concentrations (Fig. 2*F*). Approaches to determine the equilibrium dissociation constant (K_d_) are unlikely to have their measurements affected by direct transfer, since ligands that prematurely dissociate during direct transfer events are likely replaced by another ligand, keeping the total concentration of complex constant. This has been discussed in greater depth for FD (6), and the conclusions are generally applicable. However, we note that the steric considerations of protein–protein competition on a ligand surface are not necessarily the same for ligand–ligand competition on a protein surface.

### Biological Relevance of Direct Transfer.

Since the potential for direct transfer between polynucleotides might be an intrinsic property of many NBPs, it is natural to speculate on the potential for flux through a direct transfer versus classic pathway in vivo. Our Fig. 5 plot indicates that NBPs have an average k_θ_/k_−1_ ≈ 10^5^ M^−1^, which suggests that flux through a direct transfer versus classic pathway becomes comparable around low-micromolar concentrations of free ligand. One context where this could be relevant is in a cell nucleus, where the concentration of RNA is ~1 mM in terms of ribonucleotides (~50 µM of 20-nt RNA segments) (55) and the DNA concentration is ~20 mM in terms of deoxyribonucleotides (~170 µM of 60-bp dsDNA segments) (56). Alternatively, this threshold could be achieved by modulating local effective molarity, for example via biological condensates or liquid–liquid phase-separated granules. Finally, for large NBP ligands (e.g., chromatin), it is possible that neighboring intramolecular DNA-binding sites could have high enough effective molarities, by virtue of their restricted spatial proximity, for intramolecular direct transfer to occur with comparable scale to classic dissociation (57). Similarly, nascent RNA transcripts proximal to NBP target genes could have sufficiently increased effective molarity for RNA–DNA direct transfer (13). In these cases, a noteworthy biological implication is that the mean lifetime of an NBP–ligand complex may not be a fixed value, determined by the dissociation rate constant, but may instead be modulated by the concentration of other ligands in its local environment. This concept of tunable protein turnover on binding sites has been previously discussed for direct transfer-like phenomena (6, 38). In the case of intramolecular direct transfer (e.g., on chromatin), such behavior could allow proteins to conduct a more efficient “search” for their target sites (2, 38, 40). Similarly, the case of RNA–DNA direct transfer could be a strategy for RNA-binding chromatin-associated proteins to be recruited to or evicted from their gene targets (13).

While these implications are intriguing, it is premature to predict which instances of direct transfer will be biologically relevant. We acknowledge that just because direct transfer can be measured in vitro does not mean that it represents a mechanism integral to the protein’s function in vivo. In terms of both efficiency and magnitude, TREX1 exhibits average direct transfer (Fig. 5), but there is no readily apparent role for direct transfer in promoting TREX1’s degradation of DNA to prevent nucleic acid sensing (18). However, TREX1 is purportedly capable of “sliding” along DNA for efficient substrate binding and exonucleolytic degradation (24), and direct transfer could be an alternative or additional way to reduce the dimensionality of an otherwise 3D search process (12, 35). At minimum, the observation that TREX1’s flexible loop can drastically modulate its direct transfer kinetics (Fig. 4 *E* and *F*) demonstrates the flexible loop’s critical contribution to TREX1 dsDNA–binding affinity, consistent with recent studies (24). In contrast to TREX1, our concurrent studies of PRC2 (13) suggest that direct transfer may explain some of PRC2’s biological function: RNA-mediated regulation of PRC2’s histone methyltransferase activity on nucleosomes. Thus, direct transfer has an intuitive biological role for PRC2.

Finally, what in vivo approaches might be used to test the biological role of a protein’s direct transfer? Ideally, one would specifically ablate the capacity for a protein to perform direct transfer between two ligands without affecting their independent binding, presumably through mutation of the protein or polynucleotide sequences, and then compare phenotypes. However, since direct transfer appears to be mediated by protein contacts shared between ligand species (Fig. 1*B*), modulating direct transfer might necessarily perturb the ligands’ independent binding. In that case, the mutant phenotype could not be specifically attributed to direct transfer versus affinity perturbations. Similarly, since unique protein contacts between competing ligands act as “footholds” that should affect direct transfer, trying to modify shared versus unshared protein contacts to achieve similar binding affinities with discrepant direct transfer kinetics could prove problematic. However, we look forward to future advances that may allow direct in vivo interrogation of direct transfer biology.

## Materials and Methods

Streptavidin was purchased commercially; other proteins were expressed in bacteria and purified via affinity chromatography (16, 27, 33, 58). Oligonucleotides were purchased from IDT (Coralville, IA). FP-based K_d_ experiments were performed by titrating protein with 5 nM ligand (no competitor); binding curves were regressed with standard (nonquadratic) and Hill equations. FPCD experiments (Fig. 1*D*) were performed by titrating competitor into preformed protein–ligand complex and then monitoring complex dissociation over time; apparent dissociation rates for each competitor concentration were determined via single exponential regression, off-rate versus competitor data were fit with custom regression models for classic and direct transfer to determine rate constants, and the superior model was identified via the Bayesian Information Criterion (BIC) (59). For SM-TIRF, slides were prepared by conjugating FLAG-tagged TREX1 to PEG-biotin slides via biotin-labeled α-FLAG antibody and streptavidin (43). Single-label experiments were performed with 1 nM ligand and 0 or 10 µM competitor flowed onto the slide together. Dual-label experiments were performed with 1 or 10 nM of each ligand flowed onto the slide together and utilized alternating colocalization and FRET imaging conditions on the same slides and fields of view. Detailed equations, methodology, and theoretical background are provided in *SI Appendix*.

## Supplementary Material

Appendix 01 (PDF)

## Data Availability

GitHub hosts the FPalyze (60) and SMBalyze (61) R packages. The custom scripts referenced in these methods are available on GitHub (62). For the SM experiments, raw movie files and SMBalyze output files have been uploaded to Zenodo (63), with an embargo that expires in October 2023. Our pMALcPP vector and pMALcPP/MS2-CP plasmids will be deposited to Addgene. All study data are included in the article and/or *SI Appendix*.
