# Supplementary material for: Multiple RNA- and DNA-binding proteins exhibit direct transfer of polynucleotides with implications for target-site search
Source: Proc Natl Acad Sci U S A. 2023 Jun 20;120(26):e2220537120. doi: 10.1073/pnas.2220537120 (PMC10293810; doi:10.1073/pnas.2220537120)
Supplement: Supplementary file 1 — Appendix 01 (PDF) [file pnas.2220537120.sapp.pdf]

THEORETICAL BACKGROUND

Dynamic Protein-Ligand Interactions Produce Direct Transfer Kinetics

Inter-ligand competition for dynamic protein-ligand interactions (Fig. 1b) can be generalized with a reaction scheme (Fig. 1c – Complete Reaction Scheme) that is quantitatively described by a system of differential equations (Eq. 1). If the partial interaction intermediates are presumed to be highly transient (as our data suggest), such that ([EP*],[EP*P*],[EP*D*],[ED*],[ED*D*]) << ([E],[EP],[ED],[P],[D]), then the stable reactants can be modeled with a simplified reaction scheme (Fig. 1c – Simplified Reaction Scheme) and system of equations (Eq. 2). Consider, then, a two-phase reaction under this scheme (via Eq. 2) where the pre-reaction state (phase 1) is $[E_T] >> K_{dP}$, $[P_T] < [E_T]$, and $[D_T] = 0$ in equilibrium such that $[EP] \approx [P_T]$, then reaction initiation (phase 2) occurs by competitor addition such that $[EP]_0 \approx [P_T]$, $[E]_0 \approx [E_T] – [P_T]$, $[D]_0 = [D_T]$, and all other reactants are approximately zero concentration. In such a reaction, if $[D_T] >> [E_T]$ and $[P]_{0\rightarrow\infty} \approx 0$ (ligand liberated from protein-ligand complex after initiation is lost from reaction), then the reaction obeys Eq. 3.1. Similarly, if $[D_T] >> K_{dD} + [E_T]$ such that $[E]_{0\rightarrow\infty} \approx 0$ and $k_{\theta D}\,[EP]_t\,[D]_t + k_{-1P}\,[EP]_t >> k_{\theta P}\,[ED]_t\,[P]_t$, then the reaction is well approximated by Eq. 3.1. Solving the differential equation (Eq. 3.1) yields Eq. 3.2, which is a function for exponential dissociation. Thus, for $[E_T] \geq K_{dD}$ the proposed two-phase direct transfer reactions obey Eq. 4.1 with approximately exponential dissociation. If classic competition applies ($k_{\theta P} = k_{\theta D} = 0$), this reduces to Eq. 4.2.

It is evident from Eq. 2 that the value of Eq. 4 should decrease farther below its limit as $[D_T]\rightarrow 0$, due to rebinding of dissociated ligand, and Eq. 5 is an adjustment to Eq. 4 that should approximate this behavior. To test this approximation's efficacy, we simulated reactions over a range of initial competitor concentrations (via Eq. 2) using combinations of rate constant values relevant to our chosen protein-polynucleotide interactions, then used Eq. 5 to analyze the simulated reaction sets in a format mimicking our empirical experiments (see Methods). The results of our analysis (Supp. Table 1) indicate that this approach provides highly accurate rate constant determinations and model classifications for the proposed reaction scheme (Fig. 1c – Simplified Reaction Scheme), which are applicable to our experimental strategy (Fig. 1d) and binding properties of the studied NBPs.

The Proposed Mechanism for Direct Transfer Predicts Correlated Rate Constants

Though it's not explicitly indicated by our simplified reaction scheme (Fig. 1c) for direct transfer, it is notable from our complete reaction scheme (Fig. 1c) that the displacement of one ligand molecule by another should be an opportunistic process at the sub-molecular scale. Thus, our direct transfer rate constant for competitor ($k_{\theta D}$) should be well-correlated to the ligand's dissociation rate constant (k_{-1P}), the competitor's association (k_{1D}) and dissociation (k_{-1D}) rate constants, and the protein-polynucleotide interaction dynamicity (δ) from protein/polynucleotide flexibility and ligand-competitor binding overlap. Eq. 6.1 qualitatively describes this general relationship. If, across most interactions, k_{1D} varies less than k_{-1P}, then a reasonable approximation could be maintained by reconciling k_{1D} with an arbitrary constant (a_1) and Eq. 6.1 reduces to Eq. 6.2. If additionally, $k_{-1D} \approx k_{-1P}$ due to similar or homogenous ligand/competitor species in the reaction, then one can be ignored, and Eq. 6.2 further reduces to Eq. 6.3. We note that, due to these sequential assumptions, we predict that data variation from the trendline in Fig. 5 is predominantly attributable to δ with some influence from the competitor association rate constant (k_{1D}).

The HOP Score is an Informative Metric for Direct Transfer

Our Fig. 5 findings indicate that the k_θ/k_{-1} ratio of a protein may be a good metric for relative direct transfer efficiency. Rearranging Eq. 6.3, logarithmically transforming both sides of the equation, and renaming $\log_2(\delta)$ the "hand-off" proficiency (HOP) score gives a biophysical metric for this (Eq. 7). Our regression data in Fig. 5 approximates the remaining ubiquitous constant $a_2 \approx 1.2\times10^5\ M^{-1}$, which is indicative of the average k_θ/k_{-1} ratio of numerous direct transfer proteins tested to date. Thus, the HOP score can be calculated via Eq. 7 for any FPCD experiment, where HOP scores above and below zero indicate above- and below-average proficiency of a direct transfer reaction, respectively.

SUPPLEMENTAL MATERIALS & METHODS

Purification of Proteins

Recombinant human hnRNP-$U_{673-825}$ protein (RNA binding domain) with an N-terminal fusion of His-tagged Maltose Binding Protein (HisMBP) was purified by Otto Kletzien (University of Colorado Boulder, Department of Biochemistry, Batey Lab) as previously detailed (1). Protomer concentration was determined by spectroscopy with ε_{280} = 99,240 $M^{-1}cm^{-1}$.

Streptavidin was purchased (Sigma Aldrich #S0677-5MG, Lot #SLCJ1102) as dry powder, resuspended in buffer, and its active-site (protomer) concentration determined by spectroscopy with ε_{280} = 40,300 $M^{-1}cm^{-1}$.

Recombinant murine TREX1$_{1\text{-}242}$ protein (catalytic core) was overexpressed and purified as previously detailed (2). Briefly, protomers were expressed as a fusion protein using a pLM303x vector that encodes maltose-binding protein (MBP) linked N-terminally to TREX1 with a rhinovirus 3C protease (PreScission Protease) recognition site. Plasmid was transformed into *E. coli* Rosetta2(DE3) cells (Novagen) for overexpression, cells lysed with an Emulsiflex C3 homogenizer (Avestin), then protein purified via sequential amylose column chromatography, overnight protease cleavage, and phosphocellulose (p-cell) column chromatography. The reported wild-type plasmid was subjected to site-directed mutagenesis to introduce R174A/K175A mutations or a C-terminal FLAG tag (DYKDDDDK), then the plasmids' identities were validated by sequencing. The mutated plasmids were used to obtain mutant and FLAG-tagged protein similarly to wild-type protein, with the exception that mutant protein did not bind the p-cell column and was left as an MBP + TREX1 mixture. Preparations were determined via SDS-PAGE to be >95% purity. Active-site (protomer) concentrations were determined by spectroscopy with ε_{280} = 24,142 $M^{-1}cm^{-1}$ (TREX1 and TREX1-FLAG) or ε_{280} = 90,300 $M^{-1}cm^{-1}$ (MBP-TREX1^{R174A,K175A}), and the equivalency of TREX1 concentrations between wild-type and mutant preparations was validated by SDS-PAGE.

Recombinant *C. elegans* FBF-2$_{164\text{-}575}$ protein (RNA binding domain) was purified by Chen Qiu (National Institute of Environmental Health and Safety, Lab of Traci Hall) as previously described (3). Reported concentrations are for active-sites (monomer), unless otherwise indicated.

For recombinant MS2-CP^{V75E,A81G} protein (mutant without capsid assembly (4)), a pMAL-c6T vector (NEB #N0378S) was altered by site-directed mutagenesis to replace the TEV protease recognition site with a rhinovirus 3C protease (PreScission Protease) recognition site. This produced the "pMALcPP" vector, which encodes HisMBP-protein fusions. Then, we used site-directed mutagenesis to introduce the V75E and A81G mutations into a yeast expression vector containing the wild-type MS2-CP gene fragment (provided by Roy Parker Lab, University of Colorado Boulder, Department of Biochemistry). The MS2-CP^{V75E,A81G} gene fragment was subcloned into our pMALcPP vector, and we confirmed by sequencing that the resulting plasmid encoded a HisMBP–MS2-CP^{V75E,A81G} fusion with rhinovirus 3C protease (PreScission Protease) linker. Then, plasmid was transformed into *E. coli* BL21(DE3) cells (NEB). The transformed cells were used to inoculate 20 mL media (LB + 100 µg/mL ampicillin) and incubated overnight at 37°C/200 rpm until $A_{600} \approx 5.0$. The starter culture was diluted in 1 L fresh media so that $A_{600} \approx 0.1$, incubated at 37°C/200 rpm until $A_{600} \approx 0.8$ (~2 h), induced with 0.5 mM IPTG, and incubated overnight at 16°C/200rpm. Induced cells were pelleted by centrifugation (4,000G/4°C/20 min), resuspended in 50 mL Amylose A Buffer (20 mM TRIS pH 7.5 at 25°C, 200 mM NaCl, 1 mM EDTA) + 1 Pierce Protease Inhibitor Tablet (Thermo Scientific #A32965) + 50 mg lysozyme, then lysed with an Emulsiflex C3 homogenizer (Avestin) at 15,000-18,000 psi. Lysate was clarified by centrifugation (27,000x g/4°C/30 min), then supernatant was collected. A low-pressure chromatography column connected to a peristaltic pump (5 mL/min flow rate) was prepared with 5 mL amylose resin (NEB #E8021S), followed by equilibration with 50 mL Amylose A Buffer, supernatant application, washing with 300 mL Amylose A Buffer, and elution (bulk collection) with 50 mL Amylose B Buffer (Amylose A Buffer + 10 mM maltose). To the eluent was added 1.0 mg of Prescission Protease, and the solution was loaded into 10 kDa-cutoff SnakeSkin Dialysis Tubing (Thermo Scientific #68100) and dialyzed overnight at 4°C in Nickel A Buffer (50 mM NaH_2PO_4 pH 8.0 at 25°C, 300 mM NaCl, 10 mM imidazole). A low-pressure chromatography column connected to a peristaltic pump (2 mL/min flow rate) was prepared with 15 mL nickel resin (Qiagen #30230), followed by equilibration with 50 mL Nickel A Buffer, application of the dialyzed sample (w/ 5 mL fraction collection), and washing with 50 mL Nickel A Buffer (w/ 5 mL fraction collection). A_{280} of each fraction was determined, and the protein-rich fractions pooled. SDS-PAGE indicated >95% purity. Active-site (homodimer) concentrations were determined by spectroscopy with ε_{280} = 34,000 $M^{-1}cm^{-1}$.

Preparation of Ligands

All oligonucleotides were ordered from IDT (Coralville, IA), and their sequences in IDT syntax are provided (Supp. Table 2). FAM-biotin (#53606-1MG-F) and biotin (#B4501-100MG) were ordered from Sigma Aldrich. For dsDNA constructs, complementary oligonucleotides ordered from IDT were mixed at 5 µM (ligand) or 300 µM (competitor) each in annealing buffer (50 mM TRIS pH 7.5 at 25°C, 200 mM NaCl) and subjected to a thermocycler program (95°C for 10 min, 95→4°C at 0.5 °C/min, hold at 4°C) for annealing, and annealing was then confirmed via Native-PAGE. Concentrations of all ligands were confirmed spectroscopically using manufacturer-provided extinction coefficients.

Binding Buffer Compositions

BB1 is 50 mM TRIS (pH 7.5 at 25°C), 25 mM KCl, 2.5 mM $MgCl_2$, 0.1 mM $ZnCl_2$, 0.1 mg/mL BSA, 5% v/v glycerol, 2 mM 2-mercaptoethanol. Proteins = hnRNP-U and streptavidin.

BB2 (TREX1 'Standard Buffer') is 20 mM TRIS (pH 7.5 at 25°C), 5 mM $CaCl_2$, 2 mM DTT, 0.1 mg/mL BSA. Protein = TREX1.

BB3 (TREX1 'High Salt Buffer') is 20 mM TRIS (pH 7.5 at 25°C), 5 mM $CaCl_2$, 200 mM NaCl, 2 mM DTT, 0.1 mg/mL BSA. Protein = TREX1.

BB4 (TREX1 'Chelating Buffer') is 20 mM TRIS (pH 7.5 at 25°C), 5 mM $CaCl_2$, 5 mM EDTA, 2 mM DTT, 0.1 mg/mL BSA. Protein = TREX1.

BB5 is 10 mM HEPES (pH 7.5 at 25°C), 50 mM NaCl, 0.01% v/v Tween-20, 2 mM DTT, 0.1 mg/mL BSA. Protein = FBF-2.

BB6 is 100 mM TRIS (pH 7.5 at 25°C), 10 mM $MgCl_2$, 80 mM KCl, 0.1 mg/mL BSA. Protein = MS2-CP.

FP-Based K_d Determination

Pre-reaction mix was prepared with 5 nM ligand molecule in the indicated binding buffer (see Binding Buffer Compositions), then dispensed in 36 µL volumes into the wells of a 384-well black microplate (Corning #3575). Protein was prepared at 10X the reported concentrations via serial dilution in binding buffer. Binding reactions were initiated by addition of 4 µL of the respective protein concentration to the corresponding pre-reaction mix and then incubated for 30 min at room temperature. Wells with binding buffer only were also included for blanking. Fluorescence polarization readings were then taken for 30 min in 30 s intervals with a TECAN Spark microplate reader (Ex = 481 ± 20 nm, Em = 526 ± 20 nm). Each experiment had 2 or 4 technical replicates per protein concentration (as indicated), and at least three experiments were performed per protein-polynucleotide interaction. Protein concentrations are defined previously (see Purification of Proteins).

Raw data were analyzed in R v4.1.1 with the FPalyze function (FPalyze v1.3.0 package). Briefly, polarization versus time data were calculated for each reaction, the last 10 data points for each reaction were averaged to generate an equilibrium polarization value, and equilibrium polarization values were plotted as a function of protein concentration. Plot data were regressed with Eq. 9.1-2 to calculate K_d^{app} and n for the interactions. Values from regression with Eq. 9.1 are reported in Table 1, but Eq. 9.2 regression values are provided in Supp. Fig. 1a.

FP-Based Competitive Dissociation Experiments

Pre-reaction mix was prepared with 5 nM ligand molecule and protein ≥ 2x K_{dP}^{app} (at 25°C) in the indicated binding buffer (see Binding Buffer Compositions), then dispensed in 36 µL volumes into the wells of a 384-well black microplate (Corning #3575). Competitor was prepared at 10X the reported concentrations via serial dilution in binding buffer or the respective carrier polynucleotide (Table 1) at a concentration equal to the highest competitor concentration. Pre-reaction mix and competitor dilutions were then incubated at the indicated temperature until thermal and binding equilibrium (4°C/90 min, 25°C/30 min, or 37°C/30 min). Competitive dissociation reactions were initiated by addition of 4 µL of the respective competitor concentration to the corresponding pre-reaction mix, then fluorescence polarization readings were immediately taken (the delay between initiation of the first reactions and the first polarization reading was ~90 s) at the indicated temperature (4/25/37°C) for 120 min (the streptavidin-biotin dissociation rate was so slow that the reactions had to be extended to 24 h with the plate placed in a humidity cassette to mitigate evaporation) in 30 s intervals with a TECAN Spark microplate reader (E_x = 481 ± 20 nm, E_m = 526 ± 20 nm). Each experiment had 4 technical replicates per competitor concentration, and at least three experiments were performed per protein-polynucleotide interaction unless otherwise indicated. Specific protein concentrations used were (Protein–Prey = [Protein]): TREX1^{R174A,K175A}–ds-[F]d(N)$_{60}$ = 250 nM, All Others = 100 nM. Protein concentrations were determined as described (see Purification of Proteins).

Raw data was analyzed in R v4.1.1 with the FPalyze function (FPalyze v1.3.0 package). Briefly, polarization versus time data were calculated for each reaction, the polarization data were normalized to the maximum and minimum polarization across all reactions, and each normalized reaction was fit with an exponential dissociation function (Eq. 10.1) to determine N_{min}, λ, and k_{off}^{obs} (Eq. 10.1-2). N_{min} values were plotted as a function of competitor concentration and regressed with Eq. 10.3 to determine IC_{50} for the competitors (Supp. Fig. 1b). Then k_{off}^{obs} values were plotted as a function of competitor concentration. Plot data (with background k_{off}^{obs} subtracted) were regressed via Eq. 5.1 then Eq. 5.2 with tuning parameters constrained to the Eq. 5.1 solutions, and the regression models were compared with the Bayesian Information Criterion (BIC) (5). Rate constants (k_{-1P} and/or $k_{\theta D}$) were reported from the best-performing regression model. If minimum polarization was not reached during competition experiments (e.g., due to a weak competitor), then it was manually defined with minimum polarization data from corresponding binding curve data (see FP-Based K_d Determination).

FP-Based Stoichiometry Experiments

Binding curve experiments were performed as described above (see FP-Based K_d Determination), with a few exceptions. (1) The ligand concentration was 250 nM. (2) Single experiments were performed with 4 technical replicates. (3) Protein concentrations were defined by functional units (TREX1 = homodimer, FBF-2 = monomer, MS2-CP = homodimer) instead of active-sites. (4) Plot data were regressed with Eq. 11 to determine ligand-protein stoichiometry.

Accuracy Metrics for Rate Constant Determination Strategy

Direct transfer reactions (Eq. 2) were simulated and analyzed in R v4.1.1 with a custom script (see Software, Data, and Materials Availability). Briefly, reaction time (t), $[E_T]$, $[P_T]$, $[D_T]$, K_{dP}, K_{dD}, k_{-1P}, k_{-1D}, $k_{\theta P}$, $k_{\theta D}$, start-read delay time (t_0), read frequency (Δt), coefficient of variation versus $[EP]_0$ to use for Gaussian error (σ), number of replicate data sets to generate (N), and whether to provide the true baseline signal during analysis were user-provided. Then, initial conditions

were calculated via Eq. 12, the system of differential equations (Eq. 2) was solved by numerical integration, simulated reactions were sampled (t_0 and Δt) to mimic instrument readings, sampled reaction values were used to simulate numerous (N) erred (σ) data sets with 4 technical replicates each, each simulated data set was analyzed with the FPalyze function (FPalyze v1.3.0 package), and mean ± SD and model assignment accuracy (via BIC) were determined across successfully analyzed data sets. To automate analyses, FPalyze made unsupervised estimates of Eq. 5 parameters to guide regression. Consequently, regression sometimes failed due to inadequate estimates, and data sets had to be excluded from accuracy metric calculations. By default, t = 120 min, $[E_T]$ = 3x K_{dP}, $[P_T]$ = 5 nM, $[D_T]$ = 10x($2^0,2^{-1},2^{-2},2^{-3},2^{-4},2^{-5},2^{-6},2^{-7},2^{-8},2^{-9},2^{-10}$,0) µM, t_0 = 90 s, Δt = 30 s, σ = 0.05, N = 20, and true baseline signal was not provided to FPalyze.

TIRF Microscopy-Based Single-Molecule Experiments

PEG-biotin coated microscope slides were prepared as previously described (6). Slides were (1) washed twice with 200 µL Millipore water (mpH2O), (2) twice with 200 µL reaction buffer (BB2 + 0.05% v/v NP-40), (3) incubated for 5 min with a mix of 0.2 mg/mL streptavidin and 0.8 mg/mL BSA in reaction buffer, (4) washed twice with 200 µL reaction buffer, (5) incubated for 5 min with 50 pM (single-label) or 5/20 pM (dual-label; days A/B) biotin-tagged α-FLAG monoclonal antibody (ThermoFisher Scientific #MA1-91878-BTIN, Lot #XB341977) in reaction buffer, (6) washed twice with 200 µL reaction buffer, (7) incubated for 5 min with 250 nM TREX1-FLAG protein in reaction buffer, and (8) washed twice with 200 µL reaction buffer. This generated slides with TREX1 conjugated to their surface.

For single-label experiments, TREX1-conjugated slides were photobleached with a 640 nm laser for ~5 min, treated with 1 nM [Cy5]d(N)$_5$ ligand ± 10 µM d(N)$_5$ competitor in imaging buffer (3 mM Trolox, 1% v/v glucose, 1 mg/mL glucose oxidase, and 0.1 mg/mL catalase in reaction buffer), then immediately imaged with 640 nm laser excitation and a red wavelength bandpass camera filter. Data herein are from two independent days of experimentation with data collection at two different power/exposure settings for at least four replicate movies per condition: A-0.2s is movies from day-1 at 200 ms exposure and ~710 µW laser power, A-0.5s is movies from day-1 at 500 ms exposure and ~230 µW laser power, B-0.2s is movies from day-2 at 200 ms exposure and ~710 µW laser power, B-0.5s is movies from day-2 at 500 ms exposure and ~230 µW laser power. Representative composite images of collected movies can be found in Supp. Fig. 5a.

For dual-label experiments, TREX1-conjugated slides were photobleached with 532 nm and 640 nm lasers for 10 min, treated with 1 or 10 nM [Cy5]d(N)$_5$ + 1 or 10 nM [Cy3]d(N)$_5$ (days A or B, respectively) in imaging buffer (3 mM Trolox, 1% v/v glucose, 1 mg/mL glucose oxidase, and 0.1 mg/mL catalase in reaction buffer), then immediately imaged. Co-localization experiments used 532 and 640 nm laser excitation with green and red wavelength bandpass camera filters, and FRET experiments used 532 nm laser excitation with green and red wavelength bandpass camera filters. Co-localization data are from two independent experiments with four replicate movies each, collected with ~710 µW (red) and ~1.25 mW (green) laser powers. Co-localization movies were 5 min at 150 ms (day A) or 200 ms (day B) exposures. FRET data were collected from the same dual-label experiments as the co-localization, each with four replicate movies collected under similar laser powers as the co-localization data. FRET movies were 5 min at 150 ms exposure (day A) or 1 min at 50 ms exposure (day B).

For single-label experiments, each movie was analyzed in R v4.1.1 with the SMBalyze v2.0.6 package. Briefly, (1) identities of the tiff stacked image files were blinded with the blind.input function, (2) the id.spots function was used to create a composite image, identify particles, and calculate signal intensity over time for each particle, and (3) the refine.particles function was used to manually validate particle traces, refine binding event selections, and export residence times for all binding events. The numbers of total events selected for residence time calculations for each condition were n = 825 (0 µM) and n = 947 (10 µM). The residence times were then unblinded and analyzed in R v4.1.1 with a custom script to calculate k_{off}^{obs} for each replicate, and to calculate k_{-1P} and $k_{\theta D}$ from k_{off}^{obs} data. Movies and RData output files from each step of analysis have been deposited to Zenodo (see Software, Data, and Materials Availability).

For dual-label experiments, each movie was analyzed in R v4.1.1 with the SMBalyze v2.0.6 package. Briefly, (1) broad-wavelength control images were used to align red and green camera images with the FRET.align function, (2) the FRET.id function was used to create composite images, identify particles, pair particles, and calculate signal intensity over time for each particle, and (3) the FRET.refine function was used to manually validate particle traces. For co-localization experiment data, the total number of apparent direct transfer events identified was n = 36 (day A) and n = 34 (day B) among 379 (day A) and 752 (day B) particle traces with dynamic binding states. For FRET experimental data, no stable FRET events were detected. Movies for all dual-label experiments described herein have been deposited to Zenodo (see Software, Data, and Materials Availability).

For photobleaching experiments, streptavidin-coated slides were treated with 10 pM [Cy5]d(N)$_5$[Bio], then imaged with 640 nm laser excitation and red wavelength bandpass camera filters. Data are from a single experiment with single movies collected at each power setting on different fields of view. Laser power settings were from 0.14-2.28 mW with 600 ms exposure. Data were analyzed in R v4.1.1 with the SMBalyze 2.0.6 package. Briefly, (1) the id.spots function was used on each tiff stacked image file to create a composite image, identify particles, and calculate signal intensity over time for each particle, and (2) the bleach.calc function was used to concurrently analyze the particle traces from each movie to calculate signal over time at each power setting, perform exponential regression on signal-time data to determine their respective k_{bleach} (Supp. Fig. 5b – left), and perform linear regression on k_{bleach}-power data (Supp. Fig. 5b – right). The final linear regression gave the relationship of Eq. 13.1. Using this data via Eq. 13.2, and assuming a TIRF penetration depth

(d) of 100 nm and cumulative antibody-TREX1 length (Δx) of 50 nm, our Fig. 3 experiments should have a corresponding photobleaching rate constant (k_b^*) of 7.5-8.3 x10^{-3} s^{-1} depending on laser power. Applying this and our Fig. 3 data to Eq. 13.3, we conclude that 5.4-19% (230 μW) or 6.0-21% (710 μW) of our apparent dissociation events should be attributable to photobleaching, depending on competitor concentration.

Rate Constant Correlation Analysis

Data sourced from these studies (Fig. 5 – A-D) were the average values (Table 1) from initial experiments (Fig. 2 and Table 1). Data sourced from concurrent studies on PRC2 (Fig. 5 – E-F) are from 25°C isotherm data in Table 1 of the companion manuscript (7). The first published data (Fig. 5 – G) are taken from Table 2 of its reference (8). The second published data (Fig. 5 – H) are calculated from Table 1 values of reference (9), where k_{-1P} is the dissociation rate at the lowest DNA concentration and $k_{\theta D}$ is the rate of change in dissociation rate between the highest and lowest DNA concentrations. The last published data (Fig. 5 – I-J) are taken from Figure 2 of reference (10).

Rate constants from the indicated sources were regressed with linear axes and a 0-intercept constraint in R v4.1.1 using the lm function (R base). Regression values were R^2 = 0.91 and m = 1.2x10^5 M^{-1} for all data (Fig. 5 – A-J), R^2 = 0.75 and m = 0.81x10^5 M^{-1} for these data only (Fig. 5 – A-D), and R^2 = 0.89 and m = 1.2x10^5 M^{-1} for published data only (Fig. 5 – G-J).

Diagram, Reaction Scheme, and Figure Generation

Diagrams were prepared with BioRender, reaction schemes were prepared with ChemDraw v21.0.0 (Perkin Elmer), tables were prepared with Word (Microsoft), graphs were prepared with R v4.1.1, protein structures were prepared in PyMOL v2.5.2 (Schrodinger), and figures were assembled in PowerPoint (Microsoft). The modeled murine TREX1 structure in Fig. 4 was taken from prior work (11).

Equations

For Eq. 1.1-10, terms are defined in Fig. 1c – Complete Reaction Scheme, equations give rates of change for indicated reactants as a function of time (t), and bracketed terms indicate concentrations.

(Eq. 1.1) $[E]_t' = k_{-1P}\,[EP^*]_t + k_{-1D}\,[ED^*]_t - k_{1P}\,[E]_t\,[P]_t - k_{1D}\,[E]_t\,[D]_t$

(Eq. 1.2) $[P]_t' = k_{-1P}\,[EP^*]_t + k_{-2P}\,[EP^*P^*]_t + k_{-4P}\,[EP^*D^*]_t - k_{1P}\,[E]_t\,[P]_t - k_{2P}\,[EP^*]_t\,[P]_t - k_{4P}\,[ED^*]_t\,[P]_t$

(Eq. 1.3) $[D]_t' = k_{-1D}\,[ED^*]_t + k_{-2D}\,[ED^*D^*]_t + k_{-4D}\,[EP^*D^*]_t - k_{1D}\,[E]_t\,[D]_t - k_{2D}\,[ED^*]_t\,[D]_t - k_{4D}\,[EP^*]_t\,[D]_t$

(Eq. 1.4) $[EP^*]_t' = k_{1P}\,[E]_t\,[P]_t + k_{-2P}\,[EP^*P^*]_t + k_{-3P}\,[EP]_t + k_{-4D}\,[EP^*D^*]_t - k_{-1P}\,[EP^*]_t - k_{2P}\,[EP^*]_t\,[P]_t - k_{3P}\,[EP^*]_t - k_{4D}\,[EP^*]_t\,[D]_t$

(Eq. 1.5) $[ED^*]_t' = k_{1D}\,[E]_t\,[D]_t + k_{-4P}\,[EP^*D^*]_t + k_{-3D}\,[ED]_t + k_{-2D}\,[ED^*D^*]_t - k_{-1D}\,[ED]_t - k_{4P}\,[ED^*]_t\,[P]_t - k_{3D}\,[ED^*]_t - k_{2D}\,[ED^*]_t\,[D]_t$

(Eq. 1.6) $[EP^*P^*]_t' = k_{2P}\,[EP^*]_t\,[P]_t - k_{-2P}\,[EP^*P^*]_t$

(Eq. 1.7) $[ED^*D^*]_t' = k_{2D}\,[ED^*]_t\,[D]_t - k_{-2D}\,[ED^*D^*]_t$

(Eq. 1.8) $[EP^*D^*]_t' = k_{4P}\,[ED^*]_t\,[P]_t + k_{4D}\,[EP^*]_t\,[D]_t - k_{-4D}\,[EP^*D^*]_t - k_{-4P}\,[EP^*D^*]_t$

(Eq. 1.9) $[EP]_t' = k_{3P}\,[EP^*]_t - k_{-3P}\,[EP]_t$

(Eq. 1.10) $[ED]_t' = k_{3D}\,[ED^*]_t - k_{-3D}\,[ED]_t$

For Eq. 2.1-5, terms are defined in Fig. 1c – Simplified Reaction Scheme, equations give rates of change for indicated reactants as a function of time (t), and bracketed terms indicate concentrations. For Eq. 2.6-10, apply Eq. 2.1-5 notation.

(Eq. 2.1) $[E]_t' = k_{-1P}\,[EP]_t + k_{-1D}\,[ED]_t - k_{1P}\,[E]_t\,[P]_t - k_{1D}\,[E]_t\,[D]_t$

(Eq. 2.2) $[P]_t' = k_{-1P}\,[EP]_t + k_{\theta D}\,[EP]_t\,[D]_t - k_{1P}\,[E]_t\,[P]_t - k_{\theta P}\,[ED]_t\,[P]_t$

(Eq. 2.3) $[D]_t' = k_{-1D}\,[ED]_t + k_{\theta P}\,[ED]_t\,[P]_t - k_{1D}\,[E]_t\,[D]_t - k_{\theta D}\,[EP]_t\,[D]_t$

(Eq. 2.4) $[EP]_t' = k_{1P}\,[E]_t\,[P]_t + k_{\theta P}\,[ED]_t\,[P]_t - k_{-1P}\,[EP]_t - k_{\theta D}\,[EP]_t\,[D]_t$

(Eq. 2.5) $[ED]_t' = k_{1D}\,[E]_t\,[D]_t + k_{\theta D}\,[EP]_t\,[D]_t - k_{-1D}\,[ED]_t - k_{\theta P}\,[ED]_t\,[P]_t$

(Eq. 2.6) $[E_T] = [E] + [EP] + [ED]$

(Eq. 2.7) $[P_T] = [P] + [EP]$

(Eq. 2.8) $[D_T] = [D] + [ED]$

(Eq. 2.9) $K_{dP} = \frac{k_{-1P}}{k_{1P}}$

(Eq. 2.10) $K_{dD} = \frac{k_{-1D}}{k_{1D}}$

For Eq. 3.1-4.2, apply Eq. 2 notation. Eq. 4 gives relative rate of change in protein-ligand complex under initial conditions. Eq. 4.1 allows EP to decay by both dissociation and direct transfer, while Eq. 4.2 represents the classical model with decay by dissociation only. See Theoretical Background for conditions of simplification.

(Eq. 3.1) $[EP]_t' = -(k_{-1P} + k_{\theta D}[D_T])[EP]_t$

(Eq. 3.2) $[EP]_t = [EP]_0\, e^{-(k_{-1P} + k_{\theta D}[D_T])\,t}$

(Eq. 4.1) $\lim_{[D_T] \gg [E_T]} \frac{-[EP]_0'}{[EP]_0} = k_{-1P} + k_{\theta D}[D_T]$

(Eq. 4.2) $\lim_{[D_T] \gg [E_T]} \frac{-[EP]_0'}{[EP]_0} = k_{-1P}$

For Eq. 5.1-2, apply Eq. 4 notation, and α and β are arbitrary tuning parameters. See Theoretical Background for conditions of simplification.

(Eq. 5.1) $\frac{-[EP]_0'}{[EP]_0} = k_{off}^{obs} \approx \frac{[D_T]^\beta}{\alpha^\beta + [D_T]^\beta} k_{-1P} + k_{\theta D}[D_T]$

(Eq. 5.2) $\frac{-[EP]_0'}{[EP]_0} = k_{off}^{obs} \approx \frac{[D_T]^\beta}{\alpha^\beta + [D_T]^\beta} k_{-1P}$

For Eq. 6.1-3, apply Eq. 2 notation, a_1 and a_2 are arbitrary constants, and δ is the relative dynamicity of a protein-polynucleotide interaction. See Theoretical Background for conditions of simplification.

(Eq. 6.1) $k_{\theta D} \approx a_1 \frac{\delta\, k_{1D}\, k_{-1P}}{k_{-1D}}$

(Eq. 6.2) $k_{\theta D} \approx a_2 \frac{\delta\, k_{-1P}}{k_{-1D}}$

(Eq. 6.3) $k_{\theta D} \approx a_2\, \delta\, k_{-1P}$

For Eq. 7, apply Eq. 6 notation, and $HOP_{P\rightarrow D}$ is the "Hand-Off" Proficiency score for direct transfer of a protein from ligand to competitor. See Theoretical Background for context.

(Eq. 7) $HOP_{P\rightarrow D} = \log_2\left(\frac{k_{\theta D}}{a_2\, k_{-1P}}\right)$

For Eq. 8, apply Eq. 2 notation, and $[X]_{eq}$ refers to the concentration of reactant X at equilibrium. See Theoretical Background for context.

(Eq. 8.1.1) $k_{\theta P}[ED]_{eq}[P]_{eq} = k_{\theta D}[EP]_{eq}[D]_{eq}$

(Eq. 8.1.2) $[E_T] = [P_T] = [D_T]$

(Eq. 8.1.3) $[E_T] = [EP]_{eq} + [ED]_{eq}$

(Eq. 8.1.4) $[P_T] = [P]_{eq} + [EP]_{eq}$

(Eq. 8.1.5) $[D_T] = [D]_{eq} + [ED]_{eq}$

For Eq. 9, apply Eq. 2 notation, FP_E is equilibrium polarization at a given $[E_T]$, FP_{max} is the maximum equilibrium polarization, FP_{min} is the minimum equilibrium polarization, $[E_T]$ is the total protein concentration, K_d^{app} is the apparent equilibrium dissociation constant, and n is the Hill coefficient.

(Eq. 9.1) $FP_E = (FP_{max} - FP_{min})\frac{[E_T]}{[E_T] + K_d^{app}} + FP_{min}$

(Eq. 9.2) $FP_E = (FP_{max} - FP_{min})\frac{([E_T])^n}{([E_T])^n + (K_d^{app})^n} + FP_{min}$

For Eq. 10.1-2, apply Eq. 2 notation, N_t is relative polarization at a given time (t), N_{min} is the minimum relative polarization, λ is the exponential rate constant, $[D_T]$ is the total competitor concentration, I_D is the equilibrium signal at a given $[D_T]$ (N_{min} for Eq. 10.1), I_{max} is the I_D at $[D_T] = 0$, I_{min} is the I_D at $[D_T] \rightarrow \infty$, IC_{50} is the concentration of $[D_T]$ at which equilibrium [EP] is halved relative to $[D_T] = 0$, h is the Hill slope, k_{off}^{obs} is the apparent dissociation rate, and DT_P (Fig. 2f, 'Proportion of Direct Transfer') is the proportion of protein transfer between ligands that proceeds through a direct versus classic transfer pathway (Fig. 1b) at a given free competitor effective molarity ([D]).

(Eq. 10.1) $N_t = (1 - N_{min})\, e^{-\lambda t} + N_{min}$

(Eq. 10.2) $I_D = I_{max} - (I_{max} - I_{min})\frac{([D_T])^h}{([D_T])^h + (IC_{50})^h}$

(Eq. 10.3) $k_{off} = (1 - N_{min})\,\lambda$

(Eq. 10.4) $$DT_P = \frac{k_{\theta D}\,[D]}{k_{\theta D}\,[D] + k_{-1P}}$$

For Eq. 11, apply Eq. 9 notation, $[P_T]$ is total ligand concentration, and S is ligand-protein stoichiometry.

(Eq. 11) $$FP_E = (FP_{max} - FP_{min})\frac{[P_T]+S\left([E_T]+K_d^{app}\right)-\sqrt{\left([P_T]+S\left([E_T]+K_d^{app}\right)\right)^2-4\,S\,[P_T][E_T]}}{2\,[P_T]} + FP_{min}$$

For Eq. 12.1-5, apply Eq. 2 notation.

(Eq. 12.1) $$[E]_0 = [E_T] - \frac{[E_T] + [P_T] + K_{dP} - \sqrt{([E_T] + [P_T] + K_{dP})^2 - 4\,[E_T]\,[P_T]}}{2}$$

(Eq. 12.2) $$[P]_0 = [P_T] - \frac{[E_T] + [P_T] + K_{dP} - \sqrt{([E_T] + [P_T] + K_{dP})^2 - 4\,[E_T]\,[P_T]}}{2}$$

(Eq. 12.3) $$[EP]_0 = \frac{[E_T] + [P_T] + K_{dP} - \sqrt{([E_T] + [P_T] + K_{dP})^2 - 4\,[E_T]\,[P_T]}}{2}$$

(Eq. 12.4) $$[D]_0 = [D_T]$$

(Eq. 12.5) $$[ED]_0 = 0$$

For Eq. 13.1-3, apply Eq. 2 notation, k_b^* is the photobleaching first-order rate constant (s^{-1}) for a $[Cy5]d(N)_5$ ligand conjugated to a streptavidin-coated slide via biotin-labeled α-FLAG antibody and FLAG-tagged TREX1 protein, W is the excitation power for the laser (watts), Δx is the additional separation between slide and fluorophore imparted by the antibody and TREX1 (nm), d is the TIRF penetration depth (nm), ε is the proportion of apparent dissociation events attributable to photobleaching, and 2.8 and $7.1x10^{-3}$ are constants determined empirically from the photobleaching experiments with $[Cy5]d(N)_5[Bio]$ adhered directly to a streptavidin-coated slide (Supp. Fig. 5b).

(Eq. 13.1) $$k_{bleach} = 2.8\ W + 7.1x10^{-3}$$

(Eq. 13.2) $$k_b^* = 2.8\ W\ e^{-\Delta x/d} + 7.1x10^{-3}$$

(Eq. 13.3) $$\varepsilon = \frac{k_b^*}{k_b^* + k_{-1P} + [D]\,k_{\theta D}} = \frac{k_b^*}{k_{off}^{obs}}$$

Accuracy Metrics for Rate Constant Determination Strategy

n	K_{dP} (nM)	K_{dD} (nM)	k_{-1P} (s^{-1})		k_{-1D} (s^{-1})	$k_{\theta P}$ ($M^{-1}s^{-1}$)	$k_{\theta D}$ ($M^{-1}s^{-1}$)		BIC_{acc} (%)
	Simulation	*Simulation*	*Simulation*	*Analysis*	*Simulation*	*Simulation*	*Simulation*	*Analysis*	*Analysis*
[a] 20	1.0	1.0	1.0×10^{-2}	1.0 ± 0.025 ($\times10^{-2}$)	1.0×10^{-2}	0	0	-33 ± 46	95 (75-100)
12	100	100	1.0×10^{-2}	0.97 ± 0.11 ($\times10^{-2}$)	1.0×10^{-2}	0	0	75 ± 98	92 (62-100)
[a,b,c] 19	1.0	1.0	1.0×10^{-4}	0.87 ± 0.079 ($\times10^{-4}$)	1.0×10^{-4}	0	0	-0.76 ± 0.40	100 (82-100)
[b,c] 16	100	100	1.0×10^{-4}	0.68 ± 0.72 ($\times10^{-4}$)	1.0×10^{-4}	0	0	-5.8 ± 3.3	6 (0.15-30)
[†] 100	10	10	1.0×10^{-3}	0.99 ± 0.021 ($\times10^{-3}$)	1.0×10^{-3}	0	0	0.93 ± 2.3	97 (91-99)
[†] 100	10	10	1.0×10^{-3}	0.99 ± 0.023 ($\times10^{-3}$)	1.0×10^{-3}	100	100	100 ± 4.0	100 (96-100)
[†] 100	10	10	1.0×10^{-3}	0.96 ± 0.048 ($\times10^{-3}$)	1.0×10^{-3}	500	500	500 ± 18	100 (96-100)
20	10	10	1.0×10^{-3}	0.97 ± 0.068 ($\times10^{-3}$)	1.0×10^{-3}	0	500	500 ± 22	100 (83-100)
20	10	10	1.0×10^{-3}	1.0 ± 0.021 ($\times10^{-3}$)	1.0×10^{-3}	500	0	0.76 ± 2.1	95 (75-100)
[a,c] 11	1	100	1.0×10^{-3}	1.2 ± 0.24 ($\times10^{-3}$)	1.0×10^{-3}	0	0	-12 ± 15	45 (17-77)
20	100	1	1.0×10^{-3}	1.0 ± 0.019 ($\times10^{-3}$)	1.0×10^{-3}	0	0	-3.8 ± 2.3	95 (75-100)
[a,c] 6	1	100	1.0×10^{-3}	1.1 ± 0.13 ($\times10^{-3}$)	1.0×10^{-3}	100	100	86 ± 13	100 (54-100)
19	100	1	1.0×10^{-3}	1.0 ± 0.043 ($\times10^{-3}$)	1.0×10^{-3}	100	100	98 ± 5.6	100 (82-100)
20	10	10	1.0×10^{-2}	1.1 ± 0.33 ($\times10^{-2}$)	1.0×10^{-4}	0	0	-69 ± 220	45 (23-68)
[b,c] 20	10	10	1.0×10^{-4}	0.83 ± 0.10 ($\times10^{-4}$)	1.0×10^{-2}	0	0	-0.084 ± 0.10	100 (83-100)
20	10	10	1.0×10^{-2}	1.1 ± 0.40 ($\times10^{-2}$)	1.0×10^{-4}	100	100	88 ± 250	90 (68-99)
20	10	10	1.0×10^{-4}	0.92 ± 0.079 ($\times10^{-4}$)	1.0×10^{-2}	100	100	100 ± 1.9	100 (83-100)
[†,d] 72	10	10	1.0×10^{-3}	0.97 ± 0.090 ($\times10^{-3}$)	1.0×10^{-3}	0	0	2.8 ± 7.2	96 (88-99)
[†,d] 44	10	10	1.0×10^{-3}	1.0 ± 0.11 ($\times10^{-3}$)	1.0×10^{-3}	100	100	100 ± 17	91 (78-97)
[†,d] 45	10	10	1.0×10^{-3}	2.1 ± 5.8 ($\times10^{-3}$)	1.0×10^{-3}	500	500	470 ± 93	87 (73-95)

[a] Simulations used [E_T] = 50 nM, due to K_{dP} < [P_T]; default was [E_T] = 3 x K_{dP}.
[b] Simulations used 10-hour reaction window, due to low k_{off}^{obs}; default was 2 hrs.
[c] Analyses used simulation-provided baseline signal for k_{off}^{obs} calculations, due to incomplete competition.
[d] Simulations used 20% measurement error; default was 5%.
[†] Simulations generated 100 data sets for analysis; default was 20.

Supplemental Table 1 – Rate Constant Approximation Strategy is Accurate Across Various Simulated Conditions. Reactions were simulated for the simplified reaction scheme (Fig. 1c) as described. Time-point sampling, replication, and Gaussian error were incorporated into each simulated reaction, the erred data for each reaction were regressed with an exponential dissociation curve to determine k_{off}^{obs}, plots of k_{off}^{obs} versus [D_T] were regressed with Eq. 5 to determine rate constant values, and then Eq. 5 solutions were compared with the Bayesian Information Criterion (BIC) to classify the reactions as classic competition or direct transfer. Values reported include the number of simulated data sets that were used (n), the predictive accuracy of the BIC (BIC_{acc}), and several previously defined rate constants (Eq. 2 & Fig. 1c – Simplified Reaction Scheme). Reported values from analysis (Analysis) of the simulated (Simulation) data sets are given as mean ± SD across the indicated number of data set replicates (n). Footnotes in the first column apply to the indicated simulation data set (whole row). Rate constant nomenclature is in Fig. 1d.

Oligonucleotides Synthesized by IDT

Identifier		Sequence (IDT Nomenclature, 5’ → 3’)
r(G$_3$A$_2$)$_4$[F]		rGrGrGrArArGrGrGrArArGrGrGrArArGrGrGrArA/36-FAM/
r(G$_3$A$_2$)$_4$		rGrGrGrArArGrGrGrArArGrGrGrArArGrGrGrArA
[F]d(N)$_5$		/56-FAM/GACTG
d(N)$_5$[F]		GACTG/36-FAM/
[Cy5]d(N)$_5$		/5Cy5/GACTG
[Cy5]d(N)$_5$[Bio]		/5Cy5/GACTG/3Bio/
[Cy3]d(N)$_5$		/5Cy3/GACTG
d(N)$_5$		GACTG
ds-[F]d(N)$_{60}$	s1	/56-FAM/GAAGTGCCCGTGACGCGCGCGACGCCAGCCGACGAAGGCGGGACCCAG AGCGCGCGCCGT
	s2	/56-FAM/ACGGCGCGCGCTCTGGGTCCCGCCTTCGTCGGCTGGCGTCGCGCGCGT CACGGGCACTTC
[F]RNA^{PUF}		/56-FAM/rArArCrArUrGrUrGrCrCrArUrA
RNA^{PUF}		rArArCrArUrGrUrGrCrCrArUrA
[F]RNA2^{MS2}		/56-FAM/rGrGrArCrArUrGrArGrGrArUrCrArCrCrCrArUrGrU
RNA1^{MS2}		rGrGrGrArCrUrGrArGrGrArUrCrArCrCrCrArGrUrCrUrArU
r(A)$_{20}$ or C$_1$		rArArArArArArArArArArArArArArArArArArArA
r(N)$_5$ or C$_2$		rGrArCrUrG
r(A)$_{10}$ or C$_3$		rArArArArArArArArArA

Supplemental Table 2 – Identities of IDT-Synthesized Polynucleotide Species. The nomenclature used for ordering oligonucleotides from IDT (Sequence) is provided for all oligonucleotides named (Identifier) in the paper.

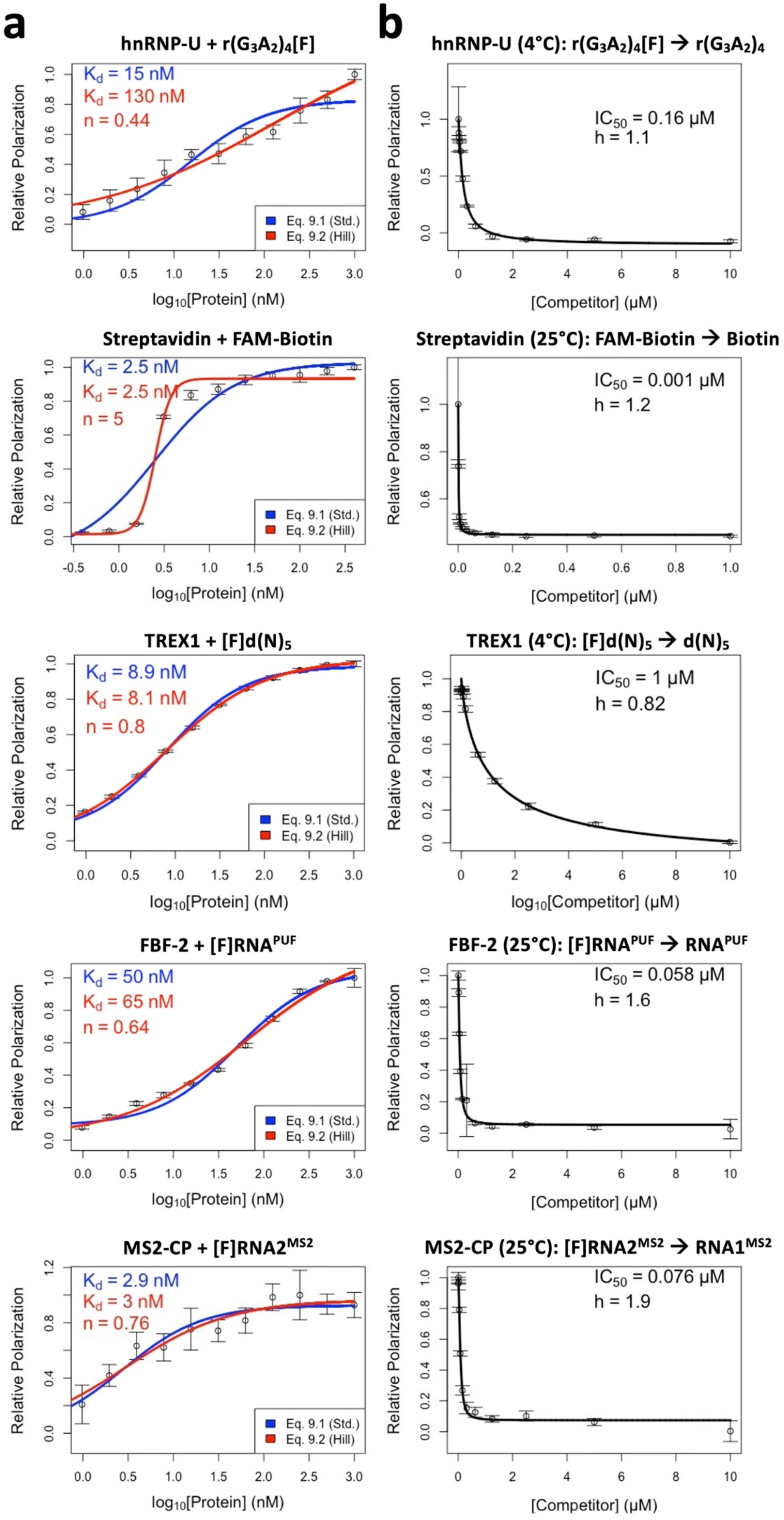
a
b
hnRNP-U + r(G3A2)4[F]
Kd = 15 nM
Kd = 130 nM
n = 0.44
Eq. 9.1 (Std.)
Eq. 9.2 (Hill)
Relative Polarization
log10[Protein] (nM)
hnRNP-U (4°C): r(G3A2)4[F] → r(G3A2)4
IC50 = 0.16 µM
h = 1.1
[Competitor] (µM)
Streptavidin + FAM-Biotin
Kd = 2.5 nM
Kd = 2.5 nM
n = 5
Streptavidin (25°C): FAM-Biotin → Biotin
IC50 = 0.001 µM
h = 1.2
TREX1 + [F]d(N)5
Kd = 8.9 nM
Kd = 8.1 nM
n = 0.8
TREX1 (4°C): [F]d(N)5 → d(N)5
IC50 = 1 µM
h = 0.82
log10[Competitor] (µM)
FBF-2 + [F]RNAPUF
Kd = 50 nM
Kd = 65 nM
n = 0.64
FBF-2 (25°C): [F]RNAPUF → RNAPUF
IC50 = 0.058 µM
h = 1.6
MS2-CP + [F]RNA2MS2
Kd = 2.9 nM
Kd = 3 nM
n = 0.76
MS2-CP (25°C): [F]RNA2MS2 → RNA1MS2
IC50 = 0.076 µM
h = 1.9

Supp. Fig. 1 – Equilibrium Binding and Competition Curves for Fig. 2 Studies. **[a]** *Equilibrium Binding Curves to Determine* K_d^{app}. Data points are mean ± SD across four reaction replicates in a representative experiment (of ≥ 3). Solid lines are regression with standard (blue) or Hill (red) binding models, and their corresponding equilibrium dissociation constant (K_d) and Hill coefficient (n) values are shown in color-coded text (Eq. 9.1-2). **[b]** *Equilibrium Competition Curves to Determine* IC_{50}. Equilibrium signal as a function of competitor concentration for Fig. 2 data. Data points are mean ± SD across four reaction replicates from the same representative experiments in Fig. 2. Solid lines are regression fits with Eq. 10.3 to determine the 50% inhibition competitor concentration (IC_{50}) and Hill slope (h) values. Standard regression values are reported in Table 1, and ligand nomenclature is defined in Supp. Table 2. See Methods for more details.

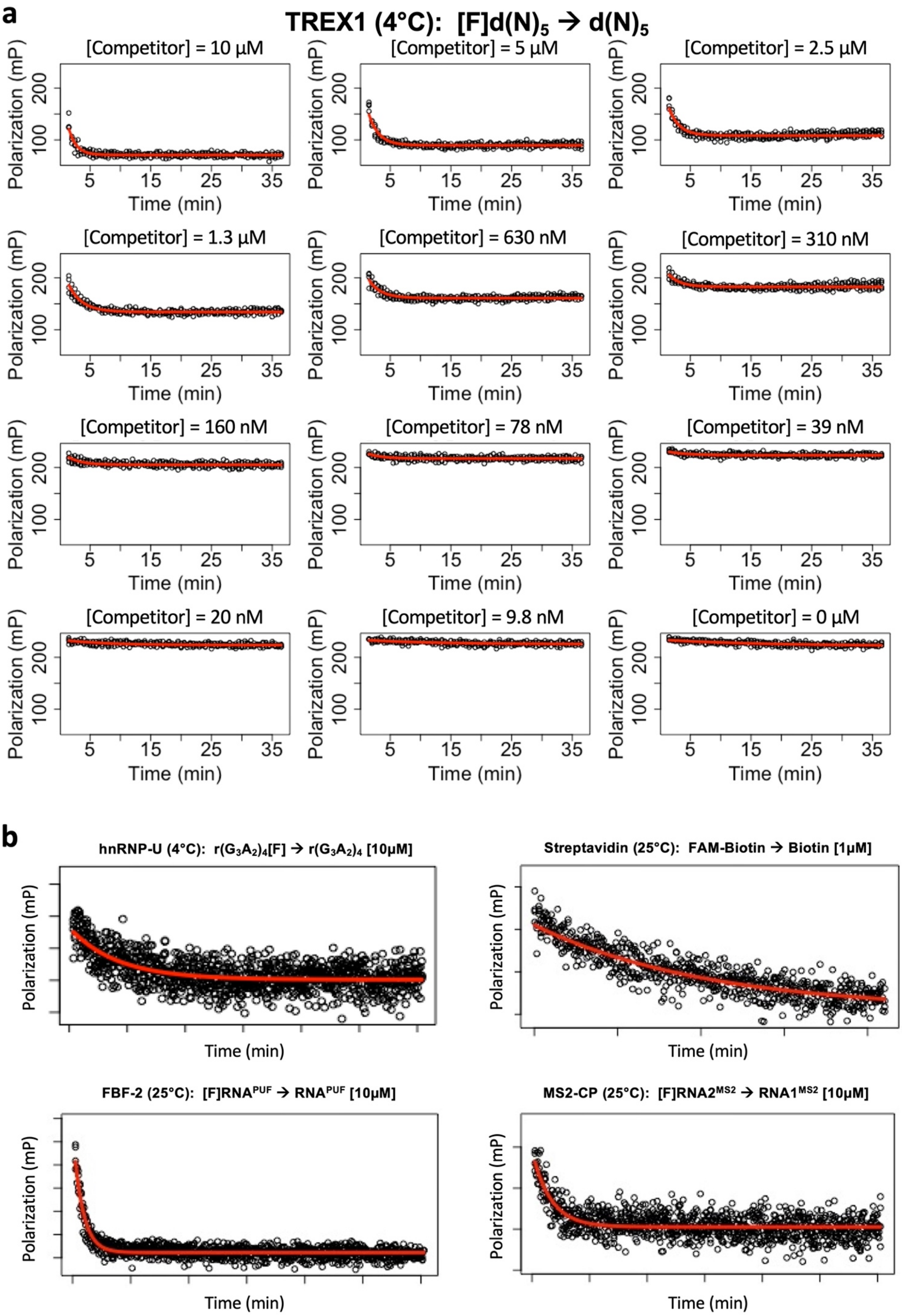
a
TREX1 (4°C): [F]d(N)5 → d(N)5
[Competitor] = 10 µM
[Competitor] = 5 µM
[Competitor] = 2.5 µM
[Competitor] = 1.3 µM
[Competitor] = 630 nM
[Competitor] = 310 nM
[Competitor] = 160 nM
[Competitor] = 78 nM
[Competitor] = 39 nM
[Competitor] = 20 nM
[Competitor] = 9.8 nM
[Competitor] = 0 µM
Polarization (mP)
100
200
Time (min)
5
15
25
35
b
hnRNP-U (4°C): r(G3A2)4[F] → r(G3A2)4 [10µM]
Streptavidin (25°C): FAM-Biotin → Biotin [1µM]
FBF-2 (25°C): [F]RNAPUF → RNAPUF [10µM]
MS2-CP (25°C): [F]RNA2MS2 → RNA1MS2 [10µM]
Polarization (mP)
Time (min)

Supp. Fig. 2 – Interrogated Protein-Polynucleotide Interactions Exhibit Exponential Dissociation. **[a]** *A Representative Protein-Ligand Interaction Exhibits Exponential Dissociation Across All Competitor Concentrations*. Raw data (black circles) fit with an exponential decay curve (red line) is shown for all competitor concentrations used in the corresponding experiment in Fig. 2. **[b]** *All Tested Protein-Ligand Interactions Exhibit Exponential Dissociation at the Excess Competitor Concentrations*. Raw data (black circles) fit with an exponential decay curve (red line) are shown for the highest competitor concentrations used in all corresponding experiments in Fig. 2. Time axes span 14 hours for streptavidin, and 2 hours for all others. Regression values are found in Table 1, and ligand/competitor definitions are in Supp. Table 2.

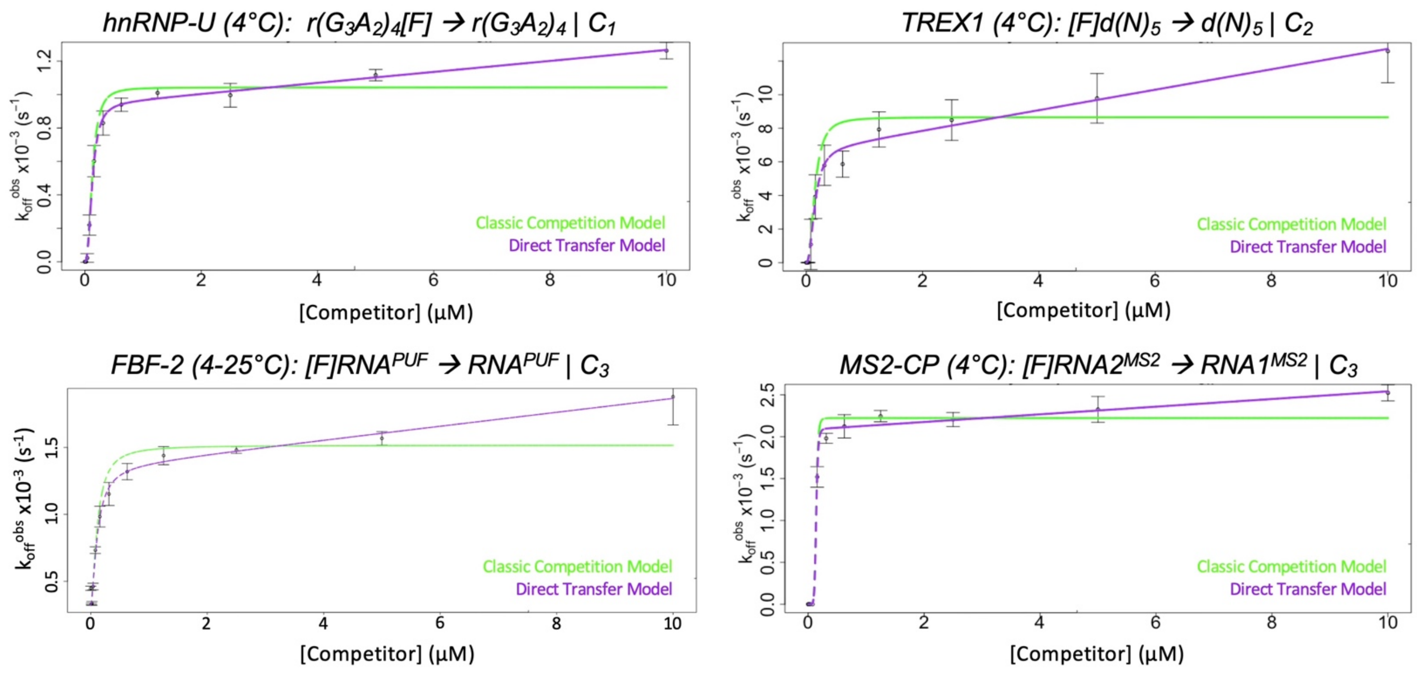

Supp. Fig. 3 – Apparent Direct Transfer is Not an Artifact of Polynucleotide Concentration. For all protein-polynucleotide interactions in Fig. 2, FPCD experiments were performed and analyzed as described (Fig. 1d) with total polynucleotide concentration kept constant via respective nonbinding carrier polynucleotides, and the final plots of apparent ligand dissociation rate (k_{off}^{obs}) versus competitor concentration are shown. Plots show best-fit regression of the data with equations describing classic competition (green lines) versus direct transfer (purple lines). Error bars are mean ± SD across four technical replicates in one experiment. The corresponding regression values can be found in Table 1, rate constant nomenclature is in Fig. 1d, and ligand/competitor definitions are in Supp. Table 2.

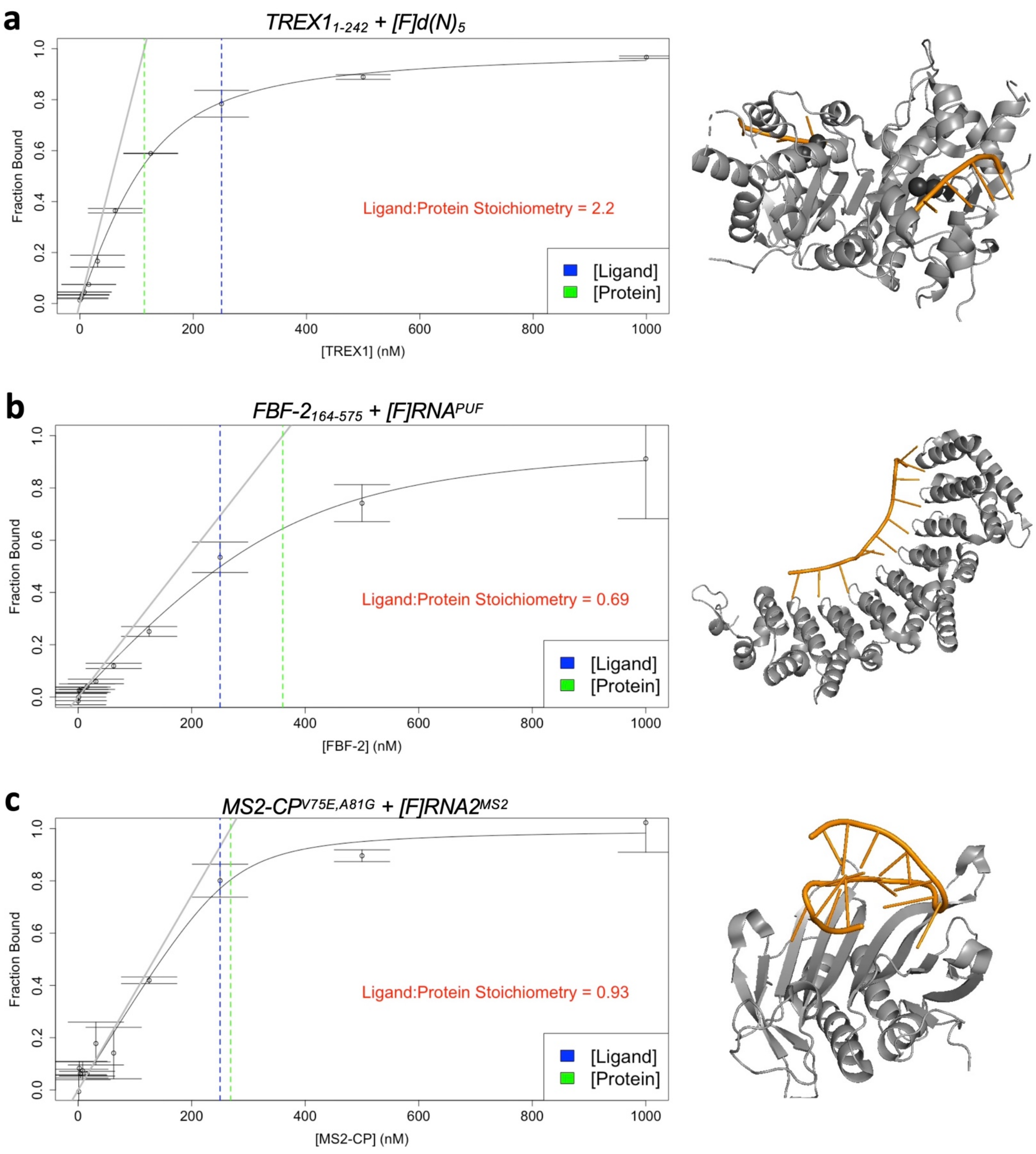

Supp. Fig. 4 – Key Protein-Polynucleotide Interactions Exhibit Expected Stoichiometry. For key protein-polynucleotide interactions, stoichiometry experiments were performed and analyzed as described, and the plots of fraction ligand bound versus protein concentration are shown alongside crystal structures of their interactions. The concentrations of protein (green) and ligand (blue) present at stoichiometric binding equilibrium are shown as vertical dashed lines. The number of ligand molecules inferred to bind to the presumed functional unit of each protein is written in red, and the value should correspond to the number of ligand molecules shown in the corresponding crystal structures. Protein concentrations are for TREX1 homodimer, FBF-2 protomer, and MS2-CP homodimer. Graphs are from single experiments, and error bars are mean ± SD across four technical replicates. Crystal structures show proteins as grey cartoons and ligands as orange cartoons/sticks. Structures in panels a-c are PDB 2OA8, 3V74, and 2C51, respectively.

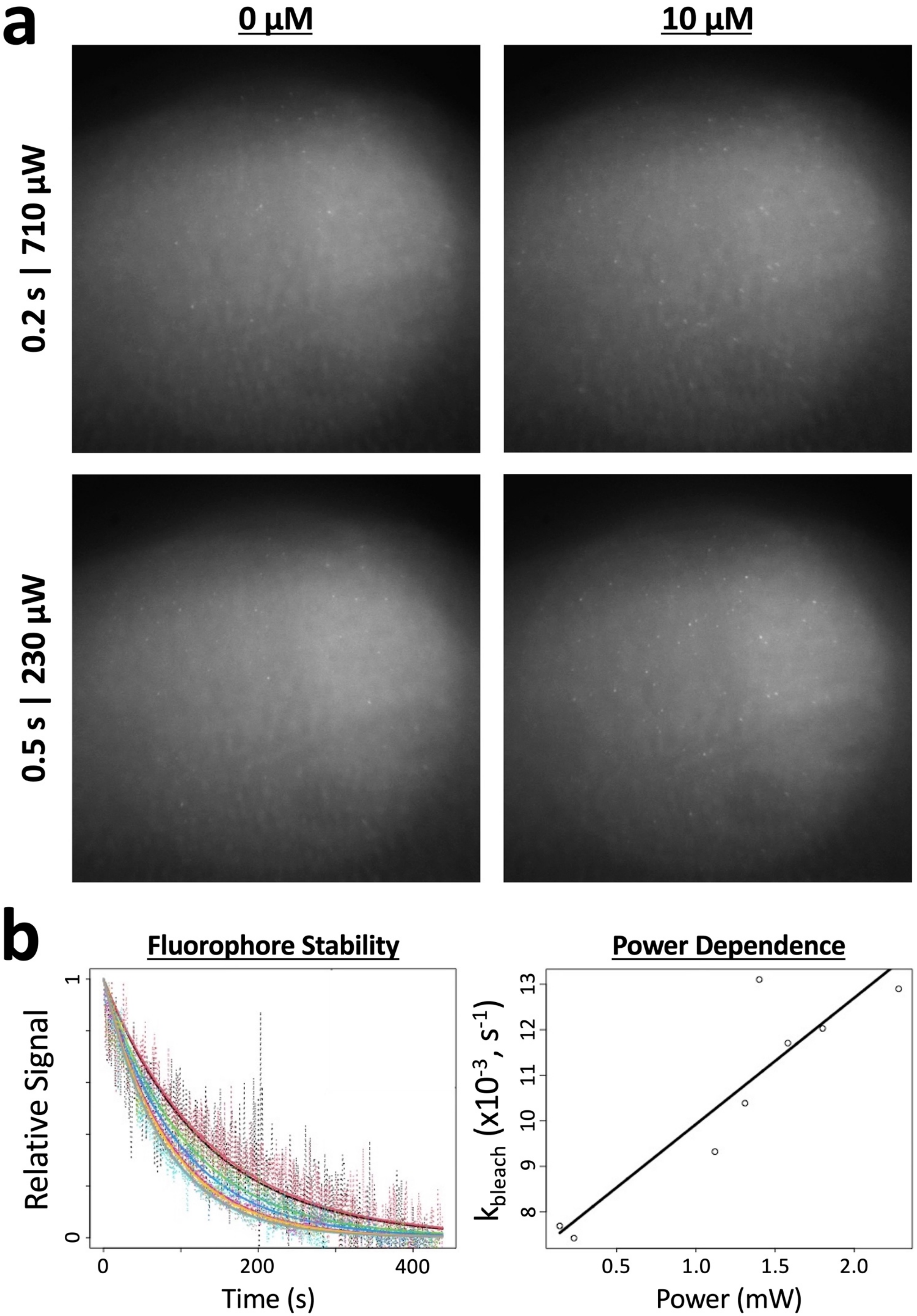

a
0 μM
10 μM
0.2 s | 710 μW
0.5 s | 230 μW
b
Fluorophore Stability
Relative Signal
Time (s)
0
1
0
200
400
Power Dependence
k_{bleach} (x10^{-3}, s^{-1})
Power (mW)
8
9
10
11
12
13
0.5
1.0
1.5
2.0

Supp. Fig. 5 – Quality Control Data for Single-Molecule TIRF Experiments. **[a]** *Representative composite images for Fig. 3 data*. Images shown are representative composites of movies collected for Fig. 3 data. Competitor conditions for images are given on the top axis, and exposure | power conditions on the left axis. **[b]** *Photobleaching kinetics for Cy5 fluorophore.* The [Cy5]d$(N)_5$ ligand used in Fig. 3 experiments was biotin labeled, adhered directly to the streptavidin-coated slides, and signal monitored over time at varying excitation power. Signal vs time curves (left) were fit with exponential regression to solve for k_{bleach} (photobleaching first-order rate constant) at each power, then the k_{bleach} vs power data (right) fit with linear regression. Linear regression indicates that $k_{bleach} = 2.8xW + 7.1x10^{-3}$ (W = power in wattage).

SUPPLEMENTAL REFERENCES

1. O. Kletzien, "Promiscuous RNA binding by the tandem RGG/RG domains of hnRNP U," University of Colorado at Boulder, United States -- Colorado. (2022) (August 17, 2022).

2. W. O. Hemphill, R. Fenske, A. R. Gooding, T. R. Cech, PRC2 direct transfer from G-quadruplex RNA to dsDNA: Implications for RNA-binding chromatin modifiers. bioRxiv (2022). https://www.biorxiv.org/content/10.1101/2022.11.30.518601v1 (accessed 17 May 2023).

3. C. Qiu, *et al.*, A crystal structure of a collaborative RNA regulatory complex reveals mechanisms to refine target specificity. *eLife* **8**, e48968.

4. K. A. LeCuyer, L. S. Behlen, O. C. Uhlenbeck, Mutants of the Bacteriophage MS2 Coat Protein That Alter Its Cooperative Binding to RNA. *Biochemistry* **34**, 10600–10606 (1995).

5. Y. Sakamoto, M. Ishiguro, G. Kitagawa, *Akaike Information Criterion Statistics*, 3rd Ed. (D. Reidel Publishing Company, 1986).

6. E. Ly, J. A. Goodrich, J. F. Kugel, Monitoring transcriptional activity by RNA polymerase II in vitro using single molecule co-localization. *Methods* **159–160**, 45–50 (2019).

7. W. O. Hemphill, R. Fenske, A. R. Gooding, T. R. Cech, PRC2 direct transfer from G-quadruplex RNA to dsDNA: Implications for RNA-binding chromatin modifiers. 2022.11.30.518601 (2022).

8. A. G. Kozlov, T. M. Lohman, Kinetic Mechanism of Direct Transfer of *Escherichia coli* SSB Tetramers between Single-Stranded DNA Molecules [†]. *Biochemistry* **41**, 11611–11627 (2002).

9. M. G. Fried, D. M. Crothers, Kinetics and mechanism in the reaction of gene regulatory proteins with DNA. *J. Mol. Biol.* **172**, 263–282 (1984).

10. J. P. Menetski, S. C. Kowalczykowski, Transfer of recA protein from one polynucleotide to another. Kinetic evidence for a ternary intermediate during the transfer reaction. *J. Biol. Chem.* **262**, 2085–2092 (1987).

11. W. O. Hemphill, T. Hollis, F. R. Salsbury, F. W. Perrino, TREX1's Homodimer Structure Has Evolved for Double-Stranded DNA Degradation. bioRxiv (2022). https://www.biorxiv.org/content/10.1101/2022.02.25.481063v2 (accessed 17 May 2023).
